# Bright light treatment counteracts stress-induced sleep alterations in mice, via a visual circuit related to the rostromedial tegmental nucleus

**DOI:** 10.1371/journal.pbio.3002282

**Published:** 2023-09-07

**Authors:** Lu Huang, Xi Chen, Qian Tao, Xiaoli Wang, Xiaodan Huang, Yunwei Fu, Yan Yang, Shijie Deng, Song Lin, Kwok-Fai So, Xingrong Song, Chaoran Ren

**Affiliations:** 1 Key Laboratory of CNS Regeneration (Ministry of Education), Guangdong Key Laboratory of Non-human Primate Research, GHM Institute of CNS Regeneration, Jinan University, Guangzhou, China; 2 Department of Anesthesiology, Guangzhou Women and Children’s Medical Center, Guangzhou Medical University, Guangzhou, China; 3 Psychology Department, School of Medicine, Jinan University, Guangzhou, China; 4 Department of Anesthesiology, Jiangmen Central Hospital, Guangdong, China; 5 Physiology Department, School of Medicine, Jinan University, Guangzhou, China; 6 Center for Brain Science and Brain-Inspired Intelligence, Guangdong-Hong Kong-Macao Greater Bay Area, Guangzhou, China; 7 Key Laboratory of Brain and Cognitive Science, Li Ka Shing Faculty of Medicine, The University of Hong Kong, Hong Kong, China; 8 Neuroscience and Neurorehabilitation Institute, University of Health and Rehabilitation Sciences, Qingdao, China; 9 Co-innovation Center of Neuroregeneration, Nantong University, Nantong, China; UMR 5292 CNRS/U1028 INSERM, FRANCE

## Abstract

Light in the environment greatly impacts a variety of brain functions, including sleep. Clinical evidence suggests that bright light treatment has a beneficial effect on stress–related diseases. Although stress can alter sleep patterns, the effect of bright light treatment on stress–induced sleep alterations and the underlying mechanism are poorly understood. Here, we show that bright light treatment reduces the increase in nonrapid eye movement (NREM) sleep induced by chronic stress through a di–synaptic visual circuit consisting of the thalamic ventral lateral geniculate nucleus and intergeniculate leaflet (vLGN/IGL), lateral habenula (LHb), and rostromedial tegmental nucleus (RMTg). Specifically, chronic stress causes a marked increase in NREM sleep duration and a complementary decrease in wakefulness time in mice. Specific activation of RMTg–projecting LHb neurons or activation of RMTg neurons receiving direct LHb inputs mimics the effects of chronic stress on sleep patterns, while inhibition of RMTg–projecting LHb neurons or RMTg neurons receiving direct LHb inputs reduces the NREM sleep–promoting effects of chronic stress. Importantly, we demonstrate that bright light treatment reduces the NREM sleep–promoting effects of chronic stress through the vLGN/IGL–LHb–RMTg pathway. Together, our results provide a circuit mechanism underlying the effects of bright light treatment on sleep alterations induced by chronic stress.

## Abstract 2

This study reveals the beneficial effects of bright light treatment on stress-induced sleep alterations and identifies a visual circuit associated with the RMTg (rostromedial tegmental nucleus) as underlying these effects.

## Introduction

Chronic stress is not only a pathogenic factor for mood disorders but also considerably affects sleep [1]. Accumulating evidence suggests that bright light treatment has a beneficial effect on both mood and sleep disorders [2–9]. However, it is unclear whether exposure to bright light can also influence sleep alterations induced by chronic stress, and the circuit mechanisms underlying the effects of chronic stress and bright light on sleep still need to be determined.

The lateral habenula (LHb) of the epithalamus encodes stressful signals [8,10–15] and directly innervates several brain regions associated with sleep regulation, including the dorsal raphe nucleus (DRN), ventral tegmental area (VTA), and rostromedial tegmental nucleus (RMTg; also known as the GABAergic tail of the VTA). Changes in neuronal activity in the LHb have been implicated in sleep regulation [10,16–21]. Notably, studies conducted in rodents have found that the LHb can receive direct inputs from the visual thalamus and that bright light can modulate neuronal activity in the LHb [8]. Thus, light signals transmitted by visual circuits related to the LHb might be crucial for the effects of bright light treatment on sleep alterations induced by chronic stress.

In this study, we identified a visual circuit connecting the ventral lateral geniculate nucleus and intergeniculate leaflet (vLGN/IGL) of the visual thalamus and RMTg. Specifically, a subset of GABA neurons in the vLGN/IGL directly innervate CaMKIIα neurons in the LHb, which in turn activate RMTg GABA neurons. The role of the vLGN/IGL-LHb-RMTg pathway in the regulation of sleep alterations induced by chronic stress was investigated using an array of brain circuit interrogation tools, including fiber photometry, optogenetics, and chemogenetics. We demonstrate that activation of the LHb-RMTg pathway is both sufficient and necessary for the nonrapid eye movement (NREM) sleep-promoting effects of chronic stress and that the vLGN/IGL-LHb-RMTg pathway is required for the ability of bright light treatment to reduce the sleep alterations induced by chronic stress.

## Results

### Chronic stress promotes NREM sleep in mice

**Fig 1:**
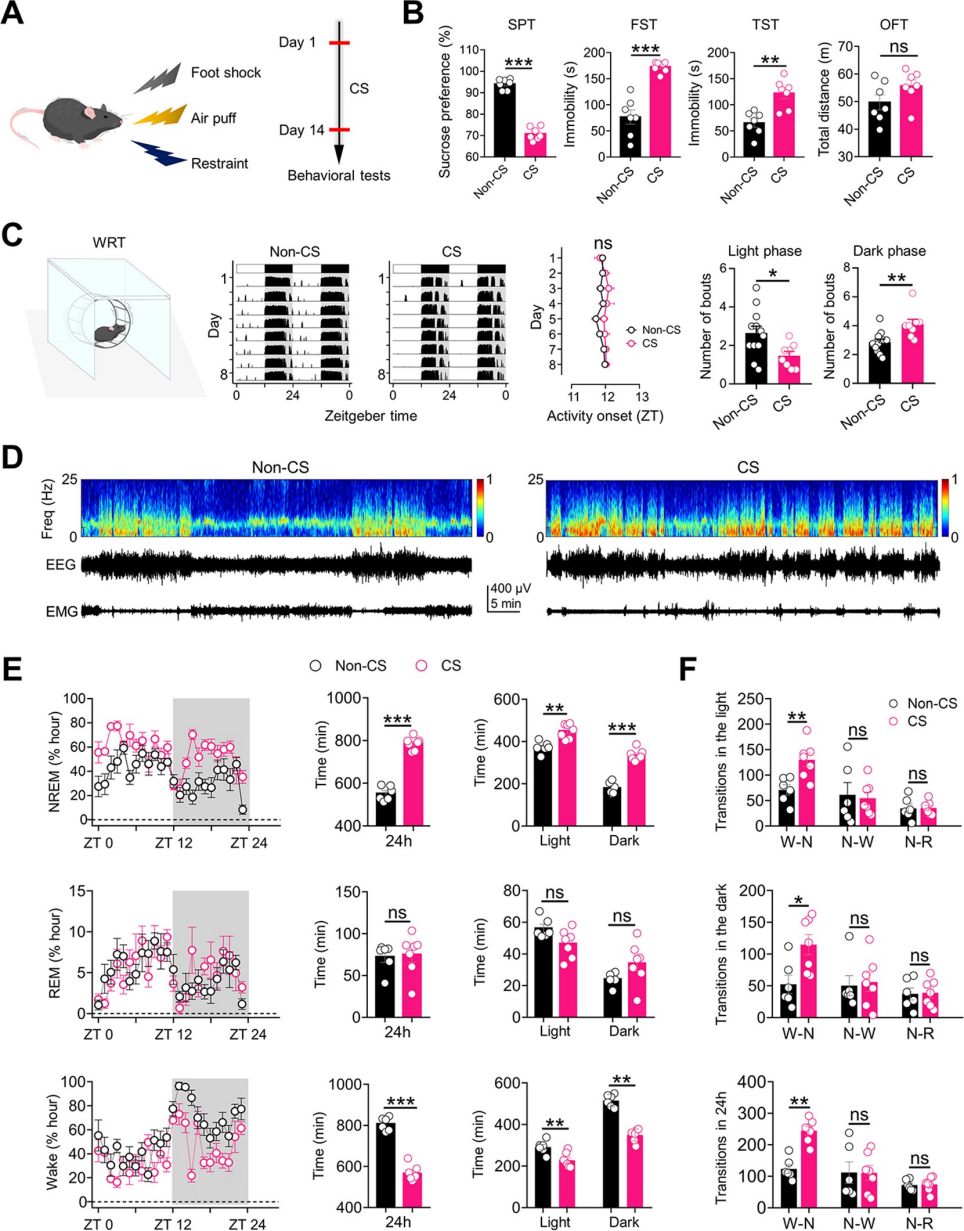
Chronic stress promotes NREM sleep in mice. ** (A)** Schematic of the experimental design. **(B)** Depressive–like behaviors in different experimental groups (*n* = 7 animals/group). Non–CS: mice that received no exposure to chronic stress stimuli (CS); CS: mice that received exposure to CS. **(C)** Left: wheel–running activities of mice in Non–CS and CS groups. Middle: activity onsets of mice in Non–CS (*n* = 12 animals) and CS (*n* = 8 animals) groups during a period of 8 days. Right: the number of activity bouts in light and dark phases of mice in Non–CS (*n* = 12 animals) and CS (*n* = 8 animals) groups. **(D)** Representative EEG spectrograms, EEG and EMG traces (recorded from ZT 1 to ZT 2) of mice in Non–CS and CS groups. **(E)** Left: time course changes of NREM and REM sleep and wakefulness of mice in Non–CS (*n* = 6 animals) and CS (*n* = 7 animals) groups. Middle: total sleep–wake amounts during the whole day (24 h) of mice in Non–CS (*n* = 6 animals) and CS (*n* = 7 animals) groups. Right: total sleep–wake amounts during light phase (ZT 0 –ZT 12) and dark phase (ZT 12 –ZT 24) of mice in Non–CS (*n* = 6 animals) and CS (*n* = 7 animals) groups. **(F)** Number of transitions between different pair of brain states during the light phase (ZT 0 –ZT 12), dark phase (ZT 12 –ZT 24), and the whole day (24 h) of mice in Non–CS (*n* = 6 animals) and CS (*n* = 7 animals) groups. W–N: Wake to NREM; N–W: NREM to Wake; N–R: NREM to REM. For all figures: one–way ANOVA with *Sidak’s* multiple comparisons test, *, *P* < 0.05; **, *P* < 0.001; ***, *P* < 0.0001; ns = no significant difference. Error bars indicate the SEM. Underlying data can be found in S1 Data. EEG, electroencephalography; EMG, electromyography; FST, forced swimming test; NREM, nonrapid eye movement; OFT, open field test; SPT, sucrose preference test; TST, tail suspension test; WRT, wheel-running test; ZT, Zeitgeber time.

To evaluate the influence of chronic stress on sleep, we first exposed mice to 2 weeks of stressful stimuli (foot shock, 20 times/day; air puff, 30 times/day; physical restraint, 1 h/day) (**Fig 1A**). Chronic stress significantly increased depressive-like behaviors in the sucrose preference test (SPT), forced swimming test (FST), and tail suspension test (TST), but did not significantly alter the locomotor activity evaluated by the open field test (OFT) (**Fig 1B**). These results suggest that 2 weeks of chronic stress increased depressive-like behaviors without affecting motor function. In addition, we evaluated the effects of exposure to chronic stress on diurnal locomotor activity assessed by the wheel-running test (WRT) (**Fig 1C**). We found that 2 weeks of chronic stress significantly reduced the fragmented light-phase activity but increased the fragmented dark-phase activity (**Fig 1C**), suggesting that chronic stress may influence the sleep/wake states of mice.

Next, we evaluated the influences of chronic stress on the sleep/wake states of mice by electromyography (EMG) and electroencephalography (EEG) after exposure to 2 weeks of chronic stress (**Fig 1D**). We found that chronic stress caused a marked increase in NREM sleep duration and a complementary decrease in wakefulness time during both the daytime and nighttime (**Fig 1D and 1E**). In contrast, the rapid eye movement (REM) sleep duration was not significantly affected by chronic stress (**Fig 1D and 1E**). To determine whether the increase in NREM sleep duration was caused by an increase in the initiation or maintenance of NREM sleep, we analyzed the transitions between different pairs of brain states. We found that chronic stress significantly increased the number of wake-NREM transitions (**Fig 1F**) without significantly affecting the number of NREM-wake and NREM-REM transitions (**Fig 1F**), indicating enhancement of the initiation but not the maintenance of NREM sleep. The above results indicate that chronic stress promotes NREM sleep in mice.

### Activation of the LHb mediates the chronic stress-induced increase in NREM sleep duration

**Fig 2:**
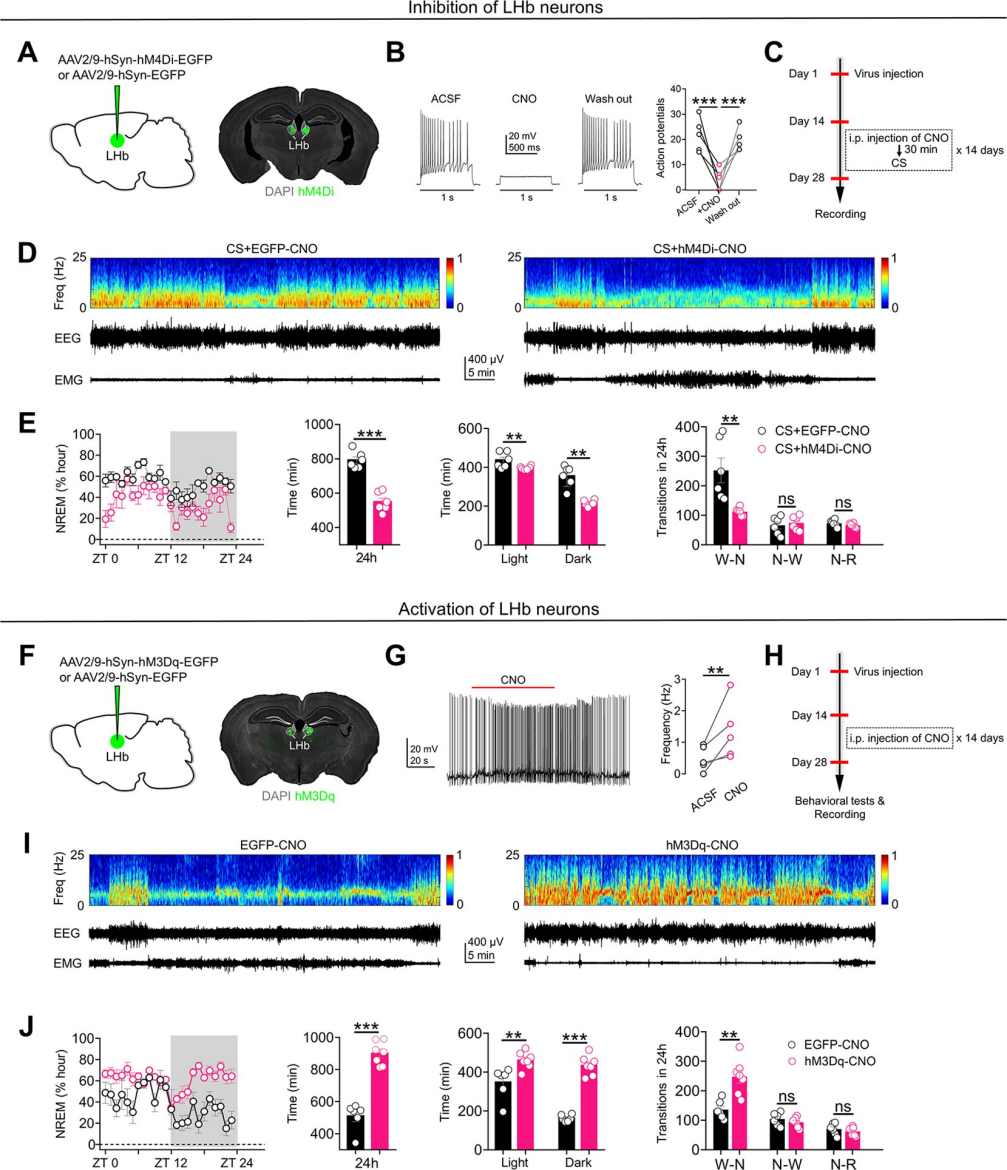
Activation of the LHb mediates the chronic stress–induced increase in NREM sleep duration. **(A)** Specific labeling of LHb neurons with hM4Di–EGFP or EGFP. **(B)** Current–evoked action potentials in a representative hM4Di infected LHb neuron recorded before, during and after CNO perfusion (10 μM). **(C)** Schematic of the experimental design. **(D)** Representative EEG spectrograms, EEG and EMG traces (recorded from ZT 1 to ZT 2) of mice in different experimental groups. All animals received exposure to chronic stress stimuli (CS) and i.p. injection of CNO (1 mg/kg). CS+EGFP–CNO: mice that received LHb injection of AAV2/9–hSyn–EGFP; CS+hM4Di–CNO: mice that received LHb injection of AAV2/9–hSyn–hM4Di–EGFP. **(E)** Left: time course changes of NREM sleep of mice in CS+EGFP–CNO and CS+hM4Di–CNO groups (*n* = 6 animals/group). Middle–left: total NREM sleep amounts during the whole day (24 h) of mice in CS+EGFP–CNO and CS+hM4Di–CNO groups (*n* = 6 animals/group). Middle–right: NREM sleep amounts during light phase (ZT 0 –ZT 12) and dark phase (ZT 12 –ZT 24) of mice in CS+EGFP–CNO and CS+hM4Di–CNO groups (*n* = 6 animals/group). Right: number of transitions between different pair of brain states during the whole day (24 h) of mice in CS+EGFP–CNO and CS+hM4Di–CNO groups (*n* = 6 animals/group). **(F)** Specific labeling of LHb neurons with hM3Dq–EGFP or EGFP. **(G)** LHb neurons expressing hM3Dq can be activated by bath application of CNO (10 μM, 100 s). **(H)** Schematic of the experimental design. **(I)** Representative EEG spectrograms, EEG and EMG traces (recorded from ZT 1 to ZT 2) of mice in different experimental groups. All animals received i.p. injection of CNO (1 mg/kg). EGFP–CNO: mice that received LHb injection of AAV2/9–hSyn–EGFP. hM3Dq–CNO: mice that received LHb injection of AAV2/9–hSyn–hM3Dq–EGFP. **(J)** Left: time course changes of NREM sleep of mice in EGFP–CNO (*n* = 6 animals) and hM3Dq–CNO (*n* = 7 animals) groups. Middle–left: total NREM sleep amounts during the whole day (24 h) of mice in EGFP–CNO (*n* = 6 animals) and hM3Dq–CNO (*n* = 7 animals) groups. Middle–right: NREM sleep amounts during light phase (ZT 0 –ZT 12) and dark phase (ZT 12 –ZT 24) of mice in EGFP–CNO (*n* = 6 animals) and hM3Dq–CNO (*n* = 7 animals) groups. Right: number of transitions between different pair of brain states during the whole day (24 h) of mice in EGFP–CNO (*n* = 6 animals/group) and hM3Dq–CNO groups (*n* = 7 animals/group). W–N: Wake to NREM; N–W: NREM to Wake; N–R: NREM to REM. For all figures: one–way ANOVA with *Sidak’s* multiple comparisons test, **, *P* < 0.001; ***, *P* < 0.0001. Error bars indicate the SEM. Underlying data can be found in S1 Data. ACSF, artificial cerebrospinal fluid; EEG, electroencephalography; EMG, electromyography; LHb, lateral habenula; NREM, nonrapid eye movement; ZT, Zeitgeber time.

Accumulating evidence suggests that the LHb encodes stressful signals and plays a pivotal role in the regulation of sleep [10,16–21]. This prompted us to explore the role of the LHb in mediating the influences of chronic stress on sleep/wake states. We induced the expression of the neuronal inhibitor DREADD hM4Di-EGFP in LHb neurons by injecting AAV2/9-hSyn-hM4Di-EGFP into the bilateral LHb (**Fig 2A**). LHb neurons expressing hM4Di-EGFP were inhibited daily via i.p. injection of CNO (1 mg/kg) for 14 days (**Figs 2B and S1A**). We found that although the long-term inhibition of RMTg-projecting LHb neurons did not significantly affect sleep/wake states in wild-type mice (**S1B–S1D Fig**), inhibiting LHb neurons during exposure to stressful stimuli reduced not only the chronic stress-induced increase in NREM sleep duration but also the effects of chronic stress on wakefulness and the number of wake-NREM transitions (**Figs 2C–2E, S1E, and S1F**). These results indicate the indispensable role played by the LHb in mediating the effects of chronic stress on sleep/wake states.

To further determine the contribution of the LHb to chronic stress-induced sleep alterations, we next evaluated the impacts of long-term activation of LHb neurons on depressive-like behaviors and sleep/wake states. We induced the expression of the neuronal activator DREADD hM3Dq-EGFP in LHb neurons by injecting AAV2/9-hSyn-hM3Dq-EGFP into the bilateral LHb (**Fig 2F**). LHb neurons were activated daily via i.p. injection of CNO (1 mg/kg) for 14 days (**Fig 2G and 2H**). We found that long-term activation of LHb neurons not only significantly increased depressive-like behaviors in the SPT, FST, and TST (**S2A Fig**) but also resulted in an increase in the duration of NREM sleep, a higher number of transitions to NREM sleep, and reduced wakefulness (**Figs 2I, 2J, S2B, and S2C**). Taken together, the above results suggest that the LHb is both necessary and sufficient for the effects of chronic stress on sleep/wake states.

### Activation of RMTg-projecting LHb neurons mimics the effects of chronic stress on sleep

**Fig 3:**
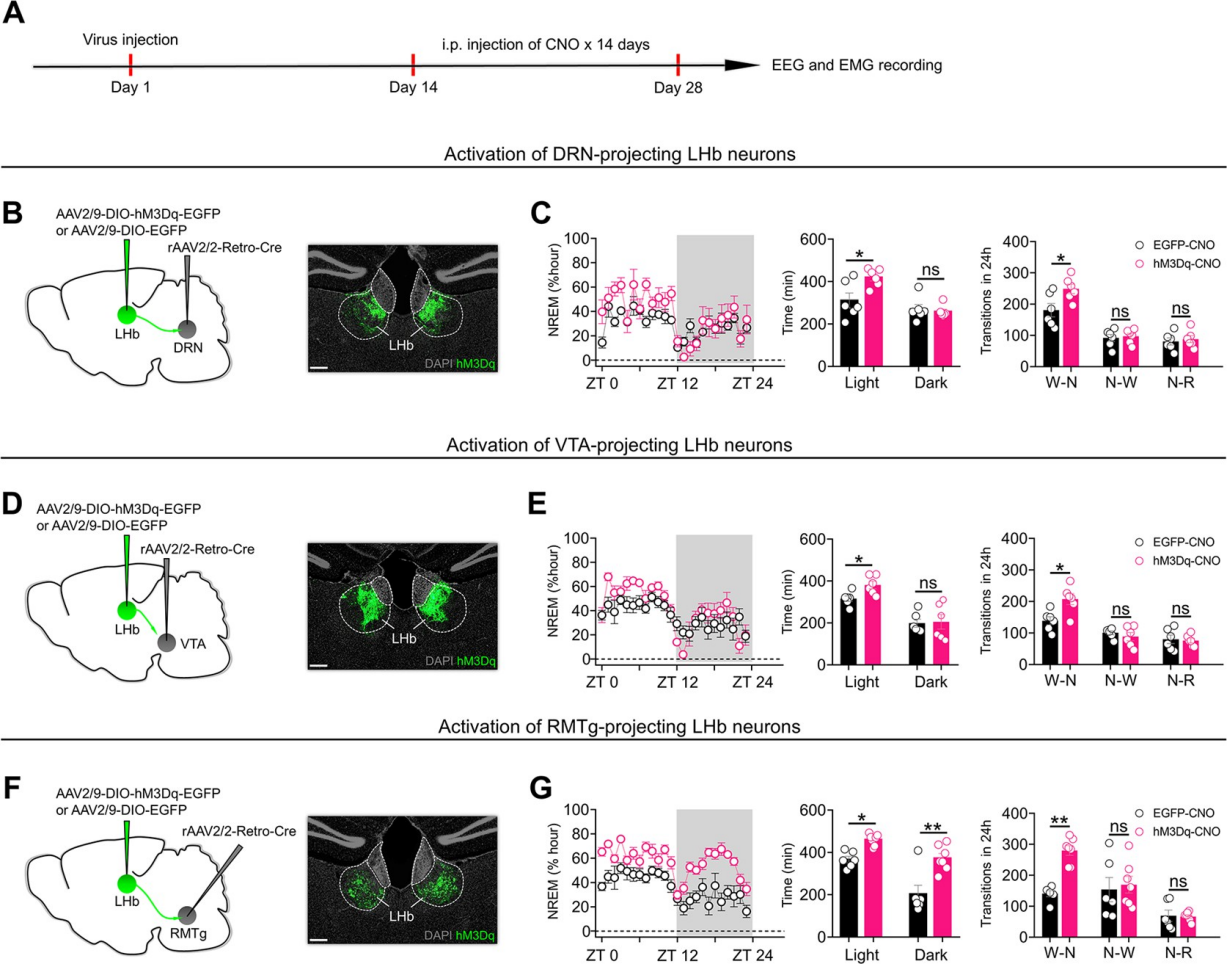
Activation of RMTg–projecting LHb neurons mimics the effects of chronic stress on sleep. **(A)** Schematic of the experimental design. **(B, D, F)** Specific labeling of DRN–projecting LHb neurons (**B**) or VTA–projecting LHb neurons (**D**) or RMTg–projecting LHb neurons (**F**) with hM3Dq–EGFP or EGFP. **(C, E, G)**. Left: time course changes of NREM sleep of mice in different experimental groups. All mice received i.p. injection of CNO (1 mg/kg). EGFP–CNO: mice that received DRN (**C**) or VTA (**E**) or RMTg (**G**) injection of rAAV2/2–Retro–Cre and LHb injection of AAV2/9–DIO–EGFP (*n* = 6 animals/group); hM3Dq–CNO: mice that received DRN (**C**, *n* = 6 animals) or VTA (**E**, *n* = 6 animals) or RMTg (**G**, *n* = 7 animals) injection of rAAV2/2–Retro–Cre and LHb injection of AAV2/9–DIO–hM3Dq–EGFP. Middle: NREM sleep amounts during light phase (ZT 0 –ZT 12) and dark phase (ZT 12 –ZT 24) of mice in EGFP–CNO and hM3Dq–CNO groups. Right: number of transitions between different pair of brain states during the whole day (24 h) of mice in EGFP–CNO and hM3Dq–CNO groups. W–N: Wake to NREM; N–W: NREM to Wake; N–R: NREM to REM. Scale bars: 200 μm (B, D, F). For all figures: one–way ANOVA test, *, *P* < 0.05; **, *P* < 0.01; ***, *P* < 0.0001. Error bars indicate the SEM. Underlying data can be found in S1 Data. DRN, dorsal raphe nucleus; LHb, lateral habenula; NREM, nonrapid eye movement; RMTg, rostromedial tegmental nucleus; VTA, ventral tegmental area; ZT, Zeitgeber time.

It is well established that LHb neurons can directly innervate several brain regions implicated in sleep regulation, including the DRN, VTA, and RMTg [10,15]. Given that previous observations suggest that DRN-projecting, VTA-projecting, and RMTg-projecting LHb neurons form largely separate circuits [8,22,23] and in light of our finding that activation of LHb neurons is indispensable for the NREM sleep-promoting effects of chronic stress, we explored which subgroup of LHb neurons might be important for the regulation of NREM sleep. We first delivered the monosynaptic retrograde transport virus rAAV2/2-Retro-Cre [24] into the DRN, VTA, or RMTg (**Fig 3B, 3D and 3F**), respectively. Next, we induced the expression of hM3Dq-EGFP specifically in DRN-projecting LHb neurons, VTA-projecting LHb neurons, or RMTg-projecting LHb neurons by injecting a Cre-dependent virus encoding hM3Dq (AAV2/9-DIO-hM3Dq-EGFP) into the LHb (**Fig 3B, 3D and 3F**). DRN-projecting LHb neurons, VTA-projecting LHb neurons, or RMTg-projecting LHb neurons were activated daily via i.p. injection of CNO (1 mg/kg) for 14 days (**Fig 3A**). We found that activation of DRN-projecting LHb neurons or VTA-projecting LHb neurons only increased the duration of NREM sleep in the daytime but not the nighttime (**Figs 3C, 3E, and S3A–S3D**). In contrast, activation of RMTg-projecting LHb neurons significantly increased the duration and initiation of NREM sleep during both the daytime and nighttime (**Figs 3G, S3E and S3F**). These results suggest that activation of RMTg-projecting LHb neurons better mimics the sleep-regulating effects of global activation of LHb neurons or chronic stress than activation of DRN-projecting LHb neurons or VTA-projecting LHb neurons.

### LHb CaMKIIα neurons activate RMTg GABA neurons through direct projections

To determine the structure of the LHb-RMTg pathway in mice, we first delivered rAAV2/2-Retro-Cre into the RMTg (**S4A Fig**). Next, a Cre-dependent virus encoding the yellow fluorescent protein eYFP (AAV2/9-DIO-eYFP) was injected into the LHb (**S4A Fig**). We found that RMTg-projecting LHb neurons labeled with eYFP were mainly located in the medial and lateral part of the LHb (**S4A Fig**), with approximately 94.64% of eYFP-labeled RMTg-projecting neurons being immunopositive for CaMKIIα (**S4A Fig**). Next, to determine the identity of RMTg neurons receiving direct LHb inputs, we delivered the monosynaptic anterograde transport virus AAV2/1-Cre [25] into the LHb and AAV2/9-DIO-eYFP into the RMTg of C57BL/6 mice (**S4B Fig**). We found that approximately 91.86% of RMTg neurons receiving direct LHb inputs were immunopositive for GABA (**S4B Fig**). These results suggest that a subset of CaMKIIα-expressing LHb neurons directly innervate GABA-expressing RMTg neurons in mice.

**Fig 4:**
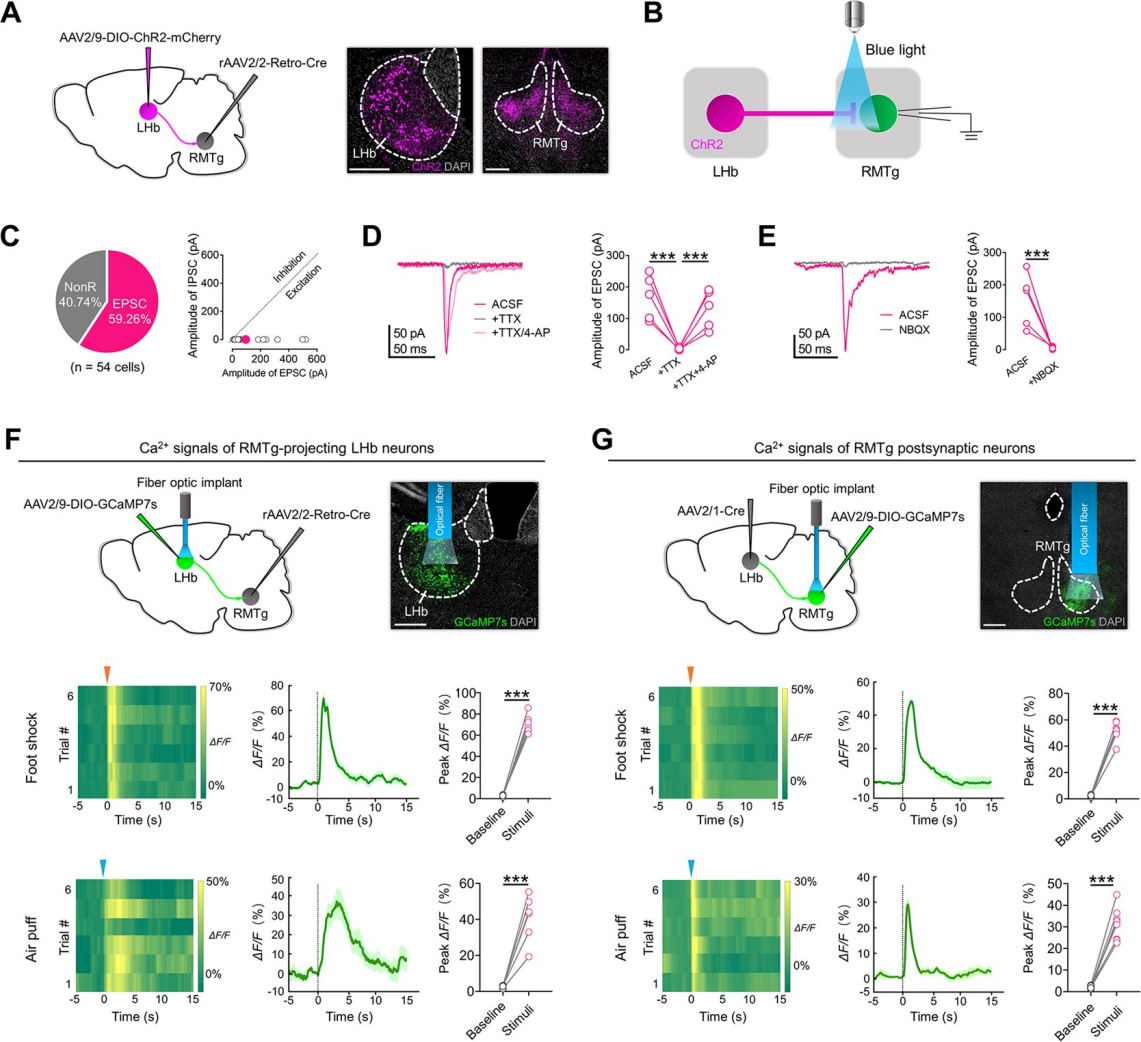
LHb CaMKIIα neurons activate RMTg GABA neurons through direct projections. **(A)** Left: scheme for specific labeling of RMTg–projecting LHb neurons with ChR2–mCherry. Right: representative images of the LHb and RMTg 2 weeks after virus injections. **(B)** Scheme for recording the optogenetically evoked postsynaptic currents in RMTg neurons. **(C)** Left: pie chart indicates whether the absolute amplitude was greater for evoked EPSCs or no response (NonR). Right: absolute amplitude of optogenetically evoked IPSCs and EPSCs in RMTg neurons (*n* = 54 cells). **(D)** Optogenetically evoked postsynaptic currents were completely blocked by the application of TTX and recovered by the application of TTX/4–AP. **(E)** Optogenetically evoked EPSCs were blocked by the application of NBQX. **(F)** Upper–left: scheme for specific infection of RMTg–projecting LHb neurons with GCaMP7s. Upper–right: representative images of the LHb 2 weeks after virus injections. Bottom–left: heat maps showing the Ca^2+^ signals evoked by foot shock (6 trials) and air puff (6 trials) in RMTg–projecting LHb neurons. Bottom–middle: averaged responses of mice in different groups. Bottom–right: stress–related stimuli significantly increased the amplitude of Ca^2+^ signals in RMTg–projecting LHb neurons (*n* = 6 animals). **(G)** Upper–left: scheme for specific infection of RMTg neurons receiving direct LHb inputs with GCaMP7s. Upper–right: representative images of the RMTg 2 weeks after virus injections. Bottom–left: heat maps showing the Ca^2+^ signals evoked by foot shock (6 trials) and air puff (6 trials) in RMTg postsynaptic neurons. Bottom–middle: averaged responses of mice in different groups. Bottom–right: stress–related stimuli significantly increased the amplitude of Ca^2+^ signals in RMTg postsynaptic neurons (*n* = 8 animals). Scale bars: 200 μm (A, F, G). For all figures: one–way ANOVA test, ***, *P* < 0.0001. Error bars indicate the SEM. Underlying data can be found in S1 Data. 4-AP, 4-aminopyridine; ACSF, artificial cerebrospinal fluid; EPSC, excitatory postsynaptic current; LHb, lateral habenula; RMTg, rostromedial tegmental nucleus; TTX, tetrodotoxin.

To determine how LHb neurons regulate neuronal activity in the RMTg, we delivered rAAV2/2-Retro-Cre into the RMTg of C57BL/6 mice and a Cre-dependent virus encoding channelrhodopsin-2 and mCherry (AAV2/9-DIO-ChR2-mCherry) into the LHb (**Fig 4A**). Next, we optogenetically activated LHb-RMTg projections and recorded postsynaptic currents from RMTg neurons (**Fig 4B**). Optogenetically activating LHb-RMTg projections evoked exclusively excitatory postsynaptic currents (EPSCs) in 59.26% of recorded neurons (**Fig 4C**). In addition, the recorded postsynaptic currents were completely blocked by the application of TTX and restored by the application of TTX/4-AP (**Fig 4D**), indicating that the postsynaptic currents recorded in RMTg neurons were elicited by direct synaptic connections between RMTg-projecting LHb neurons and the recorded RMTg neurons. Furthermore, the excitatory effects of LHb-RMTg projections could be blocked by the AMPA/kainate receptor antagonist NBQX (**Fig 4E**). The above results indicate that LHb neurons activate RMTg neurons through direct projections.

The LHb encodes stressful signals [8,10–14]. To determine whether neurons in the LHb-RMTg pathway also encode stressful signals, we first injected rAAV2/2-Retro-Cre into the RMTg and infected RMTg-projecting LHb neurons with a Cre-dependent virus encoding the Ca^2+^ indicator GCaMP7s (AAV2/9-DIO-GCaMP7s) (**Fig 4F**). Next, we used fiber photometry [26,27] to quantitatively measure stressful stimulus (foot shock and air puff)-induced changes in Ca^2+^ signals in RMTg-projecting LHb neurons (**Fig 4F**). We observed that stressful stimuli reliably evoked robust activation of RMTg-projecting LHb neurons (**Fig 4F**). Next, we used fiber photometry to quantitatively measure stressful stimulus (foot shock and air puff)-induced changes in Ca^2+^ signals in RMTg neurons receiving direct LHb inputs (**Fig 4G**). Stressful stimuli also evoked robust activation of RMTg postsynaptic neurons (**Fig 4G**). The above results indicate that the LHb-RMTg pathway encodes stressful signals.

### Activation of the LHb-RMTg pathway promotes NREM sleep

**Fig 5:**
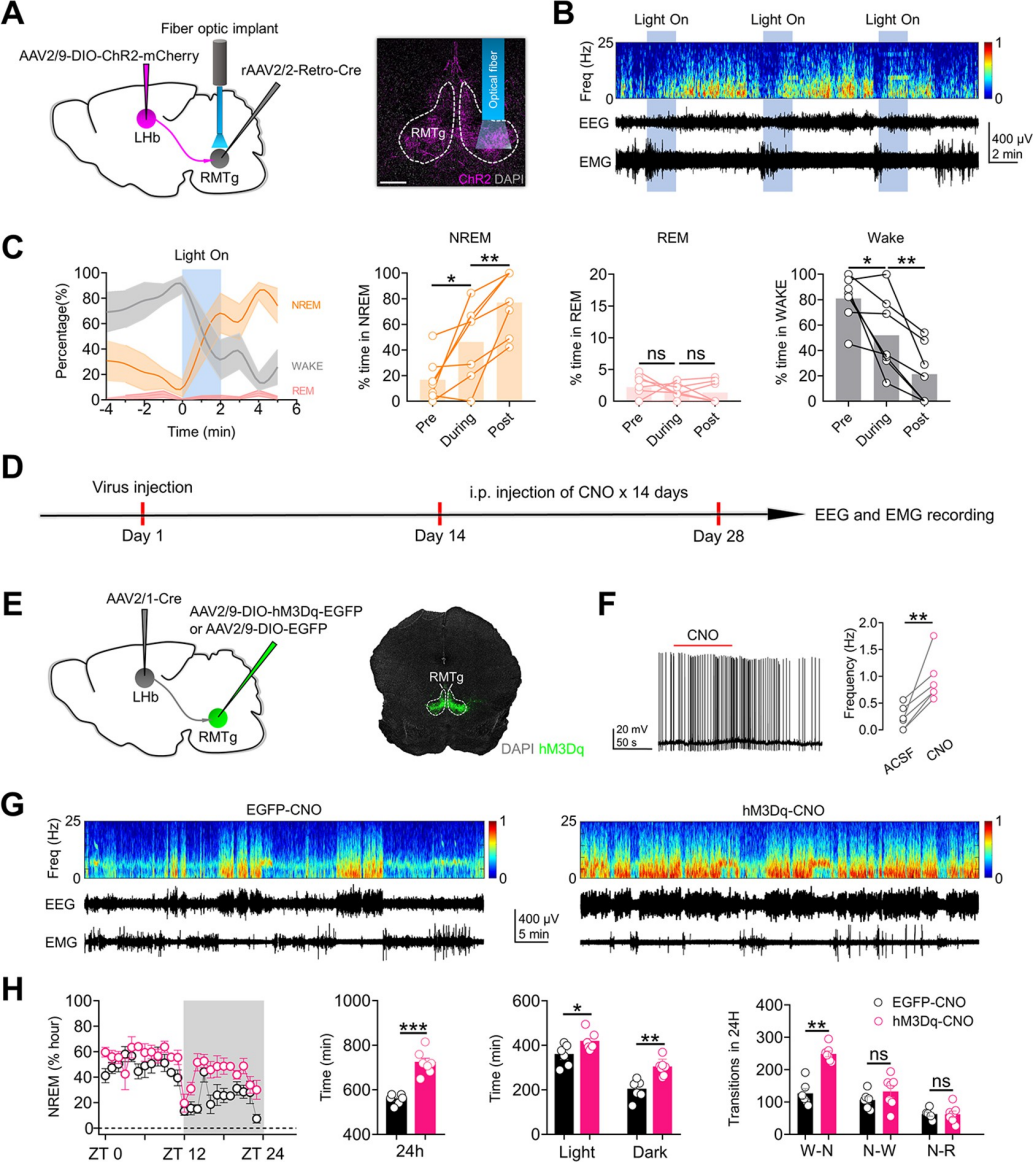
Activation of the LHb–RMTg pathway promotes NREM sleep. **(A)** Scheme for specific infection of RMTg–projecting LHb neurons with ChR2–mCherry. **(B)** An example optogenetic activation experiment in a mouse received RMTg injection of rAAV2/2–Retro–Cre and LHb injection of AAV2/9–DIO–ChR2–mCherry. Shown are the EEG spectrogram, EEG and EMG traces. Blue shading represents laser stimulation (20 Hz, 2 min). **(C)** Left: percentage of time in NREM, REM, or the wake state before, during, and after laser stimulation, averaged from 7 animals. Shading represents the 95% confidence interval, and the blue stripe indicates the laser stimulation period. Right: laser stimulation significantly increased NREM sleep and decreased wakefulness and did not significantly affect REM sleep. **(D)** Schematic of the experimental design. **(E)** Specific labeling of RMTg neurons receiving direct LHb inputs with hM3Dq–EGFP or EGFP. **(F)** RMTg postsynaptic neurons expressing hM3Dq can be activated by bath application of CNO (10 μM, 100 s). **(G)** Representative EEG spectrograms, EEG and EMG traces (recorded from ZT 1 to ZT 2) of mice in different experimental groups. All mice received LHb injection of AAV2/1–Cre and i.p. injection of CNO (1 mg/kg). EGFP–CNO: mice that received RMTg injection of AAV2/9–DIO–EGFP. hM3Dq–CNO: mice that received RMTg injection of AAV2/9–DIO–hM3Dq–EGFP. **(H)** Left: time course changes of NREM sleep of mice in EGFP–CNO (*n* = 6 animals) and hM3Dq–CNO (*n* = 7 animals) groups. Middle–left: total sleep–wake amounts during the whole day (24 h) of mice in EGFP–CNO (*n* = 6 animals) and hM3Dq–CNO groups (*n* = 7 animals). Middle–right: total sleep–wake amounts during light phase (ZT 0 –ZT 12) and dark phase (ZT 12 –ZT 24) of mice in EGFP–CNO (*n* = 6 animals) and hM3Dq–CNO (*n* = 7 animals) groups. Right: number of transitions between different pair of brain states during the whole day (24 h) of mice in EGFP–CNO (*n* = 6 animals) and hM3Dq–CNO (*n* = 7 animals) groups. W–N: Wake to NREM; N–W: NREM to Wake; N–R: NREM to REM. Scale bar: 200 μm (A). For all figures: one–way ANOVA with *Sidak’s* multiple comparisons test, *, *P* < 0.05; **, *P* < 0.001; ***, *P* < 0.0001; ns = no significant difference. Error bars indicate the SEM. Underlying data can be found in S1 Data. ACSF, artificial cerebrospinal fluid; EEG, electroencephalography; EMG, electromyography; LHb, lateral habenula; NREM, nonrapid eye movement; REM, rapid eye movement; RMTg, rostromedial tegmental nucleus; ZT, Zeitgeber time.

Given that activation of RMTg-projecting LHb neurons promotes NREM sleep (**Figs 3F, 3G, S3E and S3F**) and that the LHb can activate RMTg neurons through direct projections (**Fig 4B–4E**), we next tested whether direct activation of LHb-RMTg projections can also promote NREM sleep. We first delivered rAAV2/2-Retro-Cre into the bilateral RMTg and infected RMTg-projecting LHb neurons with AAV2/9-DIO-ChR2-mCherry (**Fig 5A**). An optic fiber was implanted above the RMTg (**Fig 5A**). Laser stimulation (20 Hz, 2 min per trial) was applied during the wake state in freely moving mice, and REM and NREM sleep, and wakefulness were classified based on EEG and EMG recordings (**Fig 5B**). We found that optogenetic activation of LHb-RMTg projections caused a marked increase in NREM sleep duration and a decrease in wakefulness time (**Fig 5B and 5C**), suggesting that short-term activation of LHb-RMTg projections promotes NREM sleep.

To further evaluate the contribution of the LHb-RMTg pathway to the regulation of sleep, we induced the expression of hM3Dq-EGFP specifically in RMTg postsynaptic neurons through injection of AAV2/1-Cre into the bilateral LHb and injection of AAV2/9-DIO-hM3Dq-EGFP into the bilateral RMTg (**Fig 5D and 5E**). RMTg neurons expressing hM3Dq-EGFP were activated daily via i.p. injection of CNO (1 mg/kg) for 14 days (**Fig 5D and 5F**). Long-term activation of RMTg neurons also significantly increased the duration of NREM sleep and the number of transitions to NREM sleep (**Figs 5G, 5H and S5**). The above results confirm that specific activation of the LHb-RMTg pathway promotes NREM sleep.

### Activation of the LHb-RMTg pathway is required for the NREM sleep-promoting effects of chronic stress

**Fig 6:**
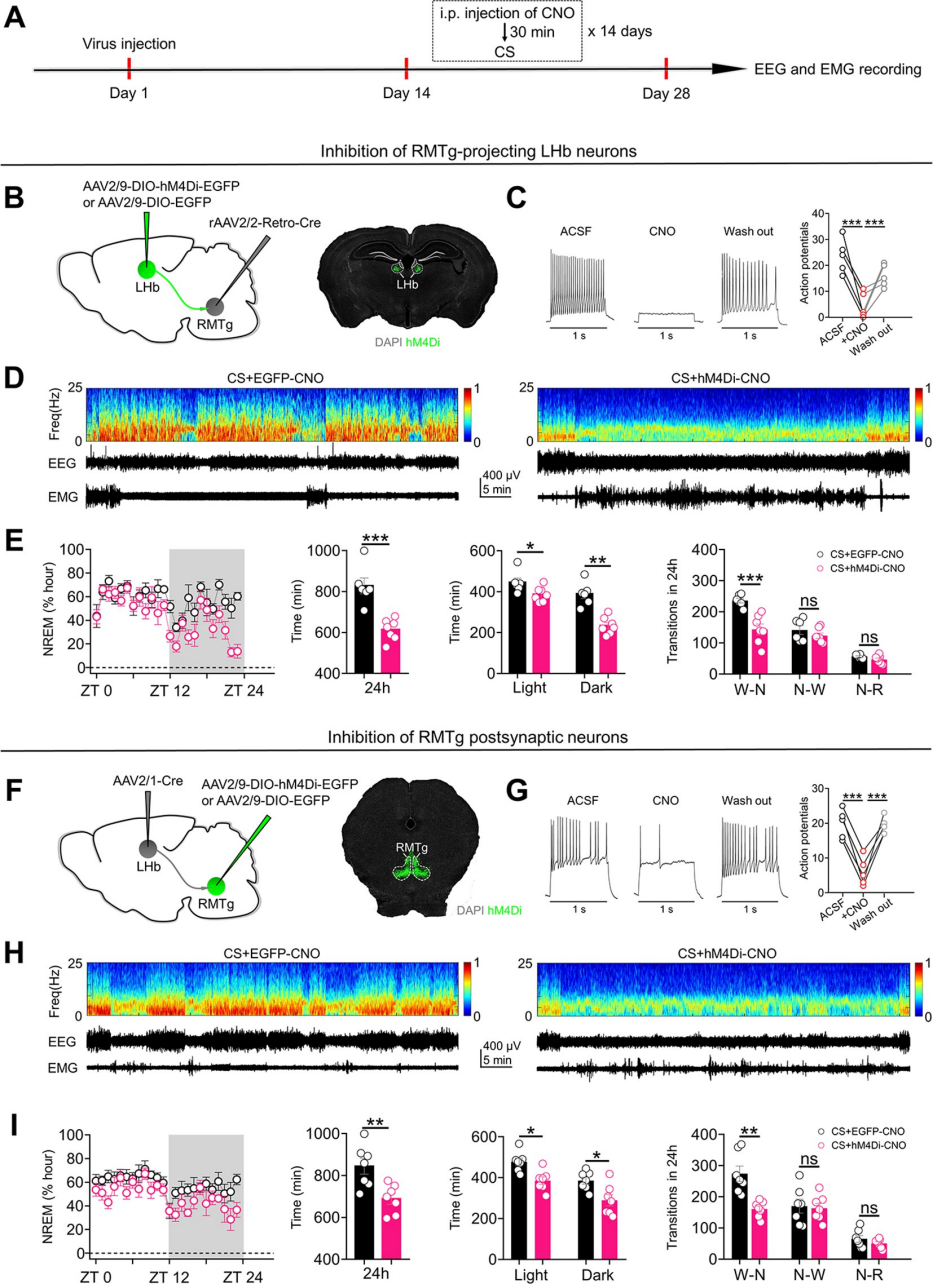
Activation of the LHb–RMTg pathway is required for the NREM sleep–promoting effects of chronic stress. **(A)** Schematic of the experimental design. **(B)** Specific labeling of RMTg–projecting LHb neurons with hM4Di–EGFP or EGFP. **(C)** Current–evoked action potentials in a representative hM4Di infected LHb neuron recorded before, during, and after CNO perfusion (10 μM). **(D)** Representative EEG spectrograms, EEG and EMG traces (recorded from ZT 1 to ZT 2) of mice in different experimental groups. All animals received exposure to chronic stress stimuli (CS), RMTg injection of rAAV2/2–Retro–Cre and i.p. injection of CNO (1 mg/kg). CS+EGFP–CNO: mice that received LHb injection of AAV2/9–DIO–EGFP. CS+hM4Di–CNO: mice that received LHb injection of AAV2/9–DIO–hM4Di–EGFP. **(E)** Left: time course changes of NREM sleep of mice in CS+EGFP–CNO (*n* = 6 animals) and CS+hM4Di–CNO (*n* = 7 animals) groups. Middle–left: total NREM sleep amounts during the whole day (24 h) of mice in CS+EGFP–CNO (*n* = 6 animals) and CS+hM4Di–CNO (*n* = 7 animals) groups. Middle–right: NREM sleep amounts during light phase (ZT 0 –ZT 12) and dark phase (ZT 12 –ZT 24) of mice in CS+EGFP–CNO (*n* = 6 animals) and CS+hM4Di–CNO (*n* = 7 animals) groups. Right: number of transitions between different pair of brain states during the whole day (24 h) of mice in CS+EGFP–CNO (*n* = 6 animals) and CS+hM4Di–CNO (*n* = 7 animals) groups. W–N: Wake to NREM; N–W: NREM to Wake; N–R: NREM to REM. **(F)** Specific labeling of RMTg neurons receiving direct LHb inputs with hM4Di–EGFP or EGFP. **(G)** Current–evoked action potentials in a representative RMTg neuron infected with hM4Di recorded before, during, and after CNO perfusion (10 μM). **(H)** Representative EEG spectrograms, EEG and EMG traces (recorded from ZT 1 to ZT 2) of mice in different experimental groups. All animals received exposure to chronic stress stimuli (CS), LHb injection of AAV2/1–Cre and i.p. injection of CNO (1 mg/kg). CS+EGFP–CNO: mice that received RMTg injection of AAV2/9–DIO–EGFP. CS+hM4Di–CNO: mice that received RMTg injection of AAV2/9–DIO–hM4Di–EGFP. **(I)** Left: time course changes of NREM sleep of mice in CS+EGFP–CNO and CS+hM4Di–CNO groups (*n* = 7 animals/group). Middle–left: total NREM sleep amounts during the whole day (24 h) of mice in CS+EGFP–CNO and CS+hM4Di–CNO groups (*n* = 7 animals/group). Middle–right: NREM sleep amounts during light phase (ZT 0 –ZT 12) and dark phase (ZT 12 –ZT 24) of mice in CS+EGFP–CNO and CS+hM4Di–CNO groups (*n* = 7 animals/group). Right: number of transitions between different pair of brain states during the whole day (24 h) of mice in CS+EGFP–CNO and CS+hM4Di–CNO groups (*n* = 7 animals). For all figures: one–way ANOVA with *Sidak’s* multiple comparisons test, *, *P* < 0.05; **, *P* < 0.001; ***, *P* < 0.0001; ns = no significant difference. Error bars indicate the SEM. Underlying data can be found in S1 Data. ACSF, artificial cerebrospinal fluid; EEG, electroencephalography; EMG, electromyography; LHb, lateral habenula; NREM, nonrapid eye movement; RMTg, rostromedial tegmental nucleus; ZT, Zeitgeber time.

Given that neurons in the LHb-RMTg pathway encode stressful signals (**Fig 4F and 4G**) and that activation of the LHb-RMTg pathway promotes NREM sleep (**Figs 3F, 3G and 5**), we postulated that activation of the LHb-RMTg pathway is important for the NREM sleep-promoting effects of chronic stress. To test this possibility, we used chemogenetics to specifically inhibit neurons in the LHb-RMTg pathway during daily exposure to stressful stimuli (**Fig 6A**). We first delivered rAAV2/2-Retro-Cre into the bilateral RMTg and infected RMTg-projecting LHb neurons with AAV2/9-DIO-hM4Di-EGFP (**Fig 6B**). RMTg-projecting LHb neurons were chemogenetically inhibited during daily exposure to stressful stimuli (**Fig 6A and 6C**). Inhibition of RMTg-projecting LHb neurons during exposure to chronic stress significantly impaired the NREM sleep-promoting effects of chronic stress (**Figs 6D, 6E, S6A and S6B**). Next, to determine whether the activation of RMTg neurons receiving direct LHb inputs is required for the NREM sleep-promoting effects of chronic stress, we injected AAV2/1-Cre into the bilateral LHb and infected postsynaptic RMTg neurons with AAV2/9-DIO-hM4Di-EGFP (**Fig 6F**). LHb postsynaptic neurons were chemogenetically inhibited during exposure to chronic stress (**Fig 6A and 6G**). We found that chemogenetic inhibition of RMTg postsynaptic neurons also impaired the NREM sleep-promoting effects of chronic stress (**Figs 6H, 6I, S6C and S6D**). Thus, activation of the LHb-RMTg pathway is required for the NREM sleep-promoting effects of chronic stress.

### Bright light treatment reduces the NREM sleep-promoting effects of chronic stress

**Fig 7:**
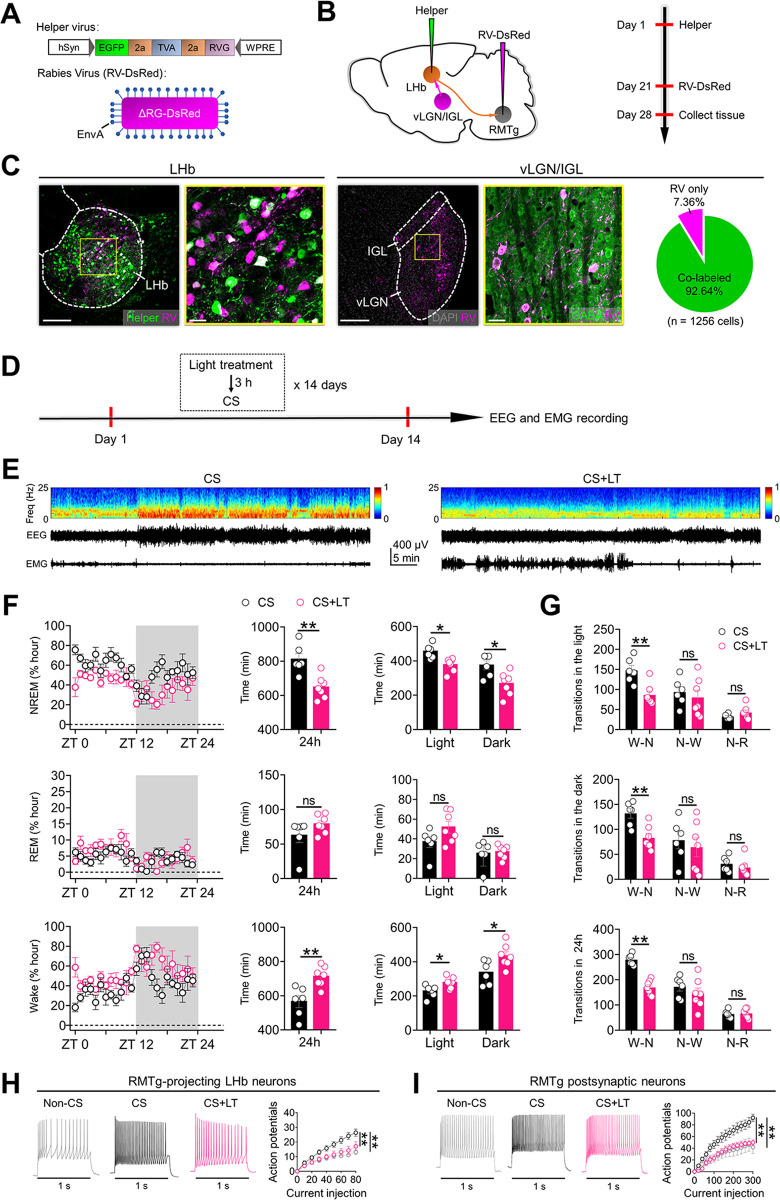
Bright light treatment reduces the NREM sleep–promoting effects of chronic stress. **(A)** Design of Helper virus and SAD–ΔG–DsRed (EnvA). **(B)** Experimental design of virus tracing in C57BL/6 mice. **(C)** Left: injection site of the LHb illustrating the location of starter cells (white). Right: a representative image of the vLGN/IGL showing RV–DsRed–labeled vLGN/IGL neurons that were immunopositive for GABA. Pie chart indicates the percentage of RV–DsRed–labeled vLGN/IGL neurons colabeled with GABA. **(D)** Schematic of the experimental design. **(E)** Representative EEG spectrograms, EEG and EMG traces (recorded from ZT 1 to ZT 2) of mice in different experimental groups. CS: mice that received exposure to chronic stress stimuli (CS). CS+LT: mice that received exposure to chronic stress stimuli and bright light treatment (3,000 lux, 2 h/day). **(F)** Left: time course changes of NREM and REM sleep and wakefulness of mice in CS (*n* = 6 animals) and CS+LT groups (*n* = 7 animals). Middle: total sleep–wake amounts during the whole day (24 h) of mice in in CS (*n* = 6 animals) and CS+LT groups (*n* = 7 animals). Right: total sleep–wake amounts during light phase (ZT 0 –ZT 12) and dark phase (ZT 12 –ZT 24) of mice in in CS (*n* = 6 animals) and CS+LT groups (*n* = 7 animals). **(G)** Number of transitions between different pair of brain states during the light phase (ZT 0 –ZT 12), dark phase (ZT 12 –ZT 24), and the whole day (24 h) of mice in CS (*n* = 6 animals) and CS+LT groups (*n* = 7 animals). W–N: Wake to NREM; N–W: NREM to Wake; N–R: NREM to REM. **(H, I)** The current–evoked action potentials recorded in RMTg–projecting LHb neurons (**H**) and RMTg neurons receiving direct LHb inputs (**I**) in different experimental groups. Non–CS: mice that did not receive exposure to chronic stress stimuli (*n* = 9 cells/group for **H**; *n* = 8 cells/group for **I**); CS: mice that receive exposure to chronic stress stimuli (*n* = 11 cells/group for **H**; *n* = 7 cells/group for **I**); CS+LT: mice that received exposure to chronic stress stimuli and bright light treatment (3,000 lux, 2 h/day) (*n* = 11 cells/group for **H**; *n* = 7 cells/group for **I**). Scale bars: 200 μm (C–LHb–left, C–vLGN/IGL–left), 20 μm (C–LHb–right, C–vLGN/IGL–right). For all figures: one–way ANOVA with *Sidak’s* multiple comparisons test, *, *P* < 0.05; **, *P* < 0.001; ns = no significant difference. Error bars indicate the SEM. Underlying data can be found in S1 Data. EEG, electroencephalography; EMG, electromyography; IGL, intergeniculate leaflet; LHb, lateral habenula; NREM, nonrapid eye movement; REM, rapid eye movement; RMTg, rostromedial tegmental nucleus; vLGN, ventral lateral geniculate nucleus; ZT, Zeitgeber time.

Bright light signals encoded by GABA neurons in the vLGN/IGL can inhibit neuronal activity in the LHb and ameliorate chronic stress-induced depressive-like symptoms [8]. We assessed whether vLGN/IGL neurons directly innervate the LHb-RMTg pathway through a modified rabies virus-based di-synaptic retrograde tracing strategy. LHb neurons were infected with AAV expressing the rabies glycoprotein and histone-tagged green fluorescent protein (Helper) (**Fig 7A and 7B**), which is required for the replication of rabies virus [28]. Next, we injected SAD-ΔG-DsRed (EnvA) into the RMTg to infect Helper^+^ RMTg-projecting LHb neurons via their presynaptic terminals (**Fig 7A and 7B**). The rabies-DsRed^+^/Glyco-EGFP^+^ double-infected LHb relay neurons (starter cells) produced infectious ΔG-rabies-DsRed that propagated transneuronally to infect vLGN/IGL neurons that formed synapses with them (**Fig 7C**). We observed that starter cells were mainly distributed in the LHb (**Fig 7C**). In the vLGN/IGL, approximately 314 neurons/animal were labeled with rabies virus (*n* = 4 animals). We found that most of the rabies virus-labeled vLGN/IGL neurons were immunopositive for GABA (approximately 92.64%), suggesting that GABA neurons in the vLGN/IGL can form di-synaptic connections with the RMTg through the LHb.

Given that activation of GABA neurons in the vLGN/IGL can inhibit the LHb [8], and inhibition of RMTg-projecting LHb neurons impairs the NREM sleep-promoting effects of chronic stress (**Figs 6D, 6E, S6A and S6B**), we tested the effects of long-term activation of LHb-projecting vLGN/IGL neurons on sleep/wake states. We delivered rAAV2/2-Retro-Cre into the bilateral LHb and infected LHb-projecting vLGN/IGL neurons with AAV2/9-DIO-hM3Dq-EGFP or AAV2/9-DIO-EGFP (**S7A Fig**). LHb-projecting vLGN/IGL neurons were activated daily via i.p. injection of CNO (1 mg/kg) for 14 days (**S7B and S7C Fig**). We found that long-term activation of LHb-projecting vLGN/IGL neurons did not significantly affect sleep/wake states in wild-type mice (**S7D–S7F Fig**). In contrast, chemogenetic activation of LHb-projecting vLGN/IGL neurons during daily exposure to stressful stimuli significantly reduced the NREM sleep-promoting effects of chronic stress (**S7G–S7J Fig**).

In light of our previous finding that bright light signals transmitted by GABA neurons in the vLGN/IGL can also inhibit the LHb [8], we hypothesized that an increase in the amount of light detected by vLGN/IGL neurons innervating the LHb-RMTg pathway can inhibit neural activity in the LHb-RMTg pathway and impair the NREM sleep-promoting effects of chronic stress. To test this possibility, during 14 days of exposure to stressful stimuli, animals received 2 h of daily bright light treatment (3,000 lux, 2 h/day) (**Fig 7D**). We found that bright light treatment significantly decreased the NREM sleep-promoting effects of chronic stress (**Fig 7E–7G**) and reduced the excitability of RMTg-projecting LHb neurons and RMTg neurons receiving direct LHb inputs (**Fig 7H and 7I**). The above results suggest that bright light treatment impairs the NREM sleep-promoting effects of chronic stress and reduces the aberrant activity of neurons in the LHb-RMTg pathway induced by chronic stress.

### Activation of the vLGN/IGL-LHb-RMTg pathway is required for the ability of bright light treatment to reduce the NREM sleep-promoting effects of chronic stress

**Fig 8:**
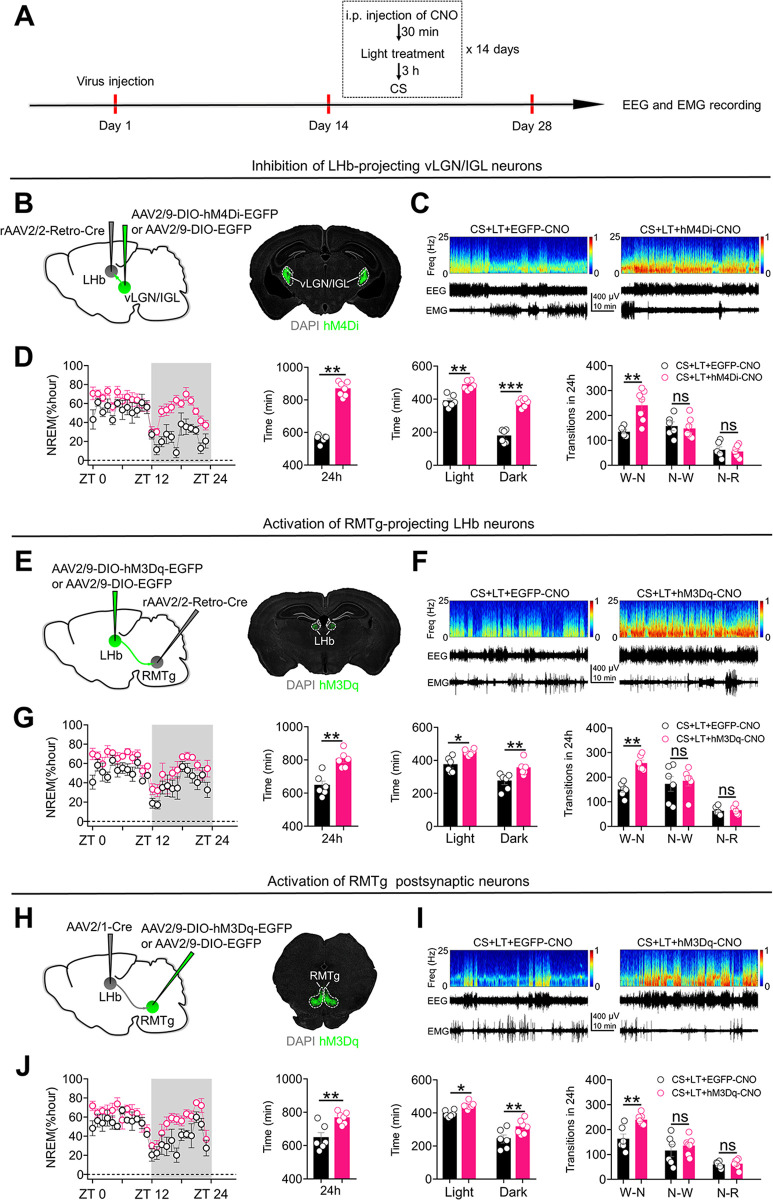
Activation of the vLGN/IGL-LHb-RMTg pathway is required for the ability of bright light treatment to reduce the NREM sleep-promoting effects of chronic stress. (**A**) Schematic of the experimental design. (**B**) Specific labeling of LHb-projecting vLGN/IGL neurons with hM4Di-EGFP or EGFP. (**C**) Representative EEG spectrograms, EEG and EMG traces (recorded from ZT 1 to ZT 2) of mice in different experimental groups. All animals received exposure to CS, LT, LHb injection of rAAV2/2-Retro-Cre and i.p. injection of CNO (1 mg/kg). CS+LT+EGFP-CNO: mice that received vLGN/IGL injection of AAV2/9-DIO-EGFP. CS+LT+hM4Di-CNO: mice that received vLGN/IGL injection of AAV2/9-DIO-hM4Di-EGFP. (**D**) Left: time course changes of NREM sleep of mice in CS+LT+EGFP-CNO (*n* = 6 animals) and CS+LT+hM4Di-CNO (*n* = 7 animals) groups; Middle-left: total NREM sleep amounts during the whole day (24 h) of mice in CS+LT+EGFP-CNO (*n* = 6 animals) and CS+LT+hM4Di-CNO (*n* = 7 animals) groups; Middle-right: NREM sleep amounts during light phase (ZT 0 – ZT 12) and dark phase (ZT 12 – ZT 24) of mice in CS+LT+EGFP-CNO (*n* = 6 animals) and CS+LT+hM4Di-CNO (*n* = 7 animals) groups. Right: number of transitions between different pair of brain states during the whole day (24 h) of mice in CS+LT+EGFP-CNO (*n* = 6 animals) and CS+LT+hM4Di-CNO (*n* = 7 animals) groups. W-N: Wake to NREM; N-W: NREM to Wake; N-R: NREM to REM. (**E**) Specific labeling of RMTg-projecting LHb neurons with hM3Dq-EGFP or EGFP. (**F**) Representative EEG spectrograms, EEG and EMG traces (recorded from ZT 1 to ZT 2) of mice in different experimental groups. All animals received exposure to chronic stress stimuli (CS), bright light treatment (LT), RMTg injection of rAAV2/2-Retro-Cre and i.p. injection of CNO (1 mg/kg). CS+LT+EGFP-CNO: mice that received LHb injection of AAV2/9-DIO-EGFP. CS+LT+hM3Dq-CNO: mice that received LHb injection of AAV2/9-DIO-hM3Dq-EGFP. (**G**) Left: time course changes of NREM sleep of mice in CS+LT+EGFP-CNO and CS+LT+hM3Dq-CNO groups (*n* = 6 animals/group); Middle-left: total NREM sleep amounts during the whole day (24 h) of mice in CS+LT+EGFP-CNO and CS+LT+hM3Dq-CNO groups (*n* = 6 animals/group); Middle-right: NREM sleep amounts during light phase (ZT 0 – ZT 12) and dark phase (ZT 12 – ZT 24) of mice in CS+LT+EGFP-CNO and CS+LT+hM3Dq-CNO groups (*n* = 6 animals/group). Right: number of transitions between different pair of brain states during the whole day (24 h) of mice in CS+LT+EGFP-CNO and CS+LT+hM3Dq-CNO groups (*n* = 6 animals/group). (**H**) Specific labeling of RMTg neurons receiving direct LHb inputs with hM3Dq-EGFP or EGFP. (**I**) Representative EEG spectrograms, EEG and EMG traces (recorded from ZT 1 to ZT 2) of mice in different experimental groups. All animals received exposure to CS, LT, LHb injection of AAV2/1-Cre and i.p. injection of CNO (1 mg/kg). CS+LT+EGFP-CNO: mice that received RMTg injection of AAV2/9-DIO-EGFP. CS+LT+hM3Dq-CNO: mice that received RMTg injection of AAV2/9-DIO-hM3Dq-EGFP. (**J**) Left: time course changes of NREM sleep of mice in CS+LT+EGFP-CNO (*n* = 6 animals) and CS+LT+hM3Dq-CNO (*n* = 7 animals) groups; Middle-left: total NREM sleep amounts during the whole day (24 h) of mice in CS+LT+EGFP-CNO (*n* = 6 animals) and CS+LT+hM3Dq-CNO (*n* = 7 animals) groups; Middle-right: NREM sleep amounts during light phase (ZT 0 – ZT 12) and dark phase (ZT 12 – ZT 24) of mice in CS+LT+EGFP-CNO (*n* = 6 animals) and CS+LT+hM3Dq-CNO (*n* = 7 animals) groups. Right: number of transitions between different pair of brain states during the whole day (24 h) of mice in CS+LT+EGFP-CNO (*n* = 6 animals) and CS+LT+hM3Dq-CNO (*n* = 7 animals) groups. For all figures: One-way ANOVA with *Sidak’s* multiple comparisons test, *P* < 0.05; **, *P* < 0.001; ***, *P* < 0.0001 ns = no significant difference. Error bars indicate the SEM. EEG, electroencephalography; EMG, electromyography; LHb, lateral habenula; NREM, nonrapid eye movement; REM, rapid eye movement; RMTg, rostromedial tegmental nucleus; ZT, Zeitgeber time.

To evaluate the role of the vLGN/IGL-LHb-RMTg pathway in the effects of bright light treatment on NREM sleep, we first induced the expression of Cre recombinase in LHb-projecting vLGN/IGL neurons by injecting rAAV2/2-Retro-Cre into the bilateral LHb and infecting LHb-projecting vLGN/IGL neurons with AAV2/9-DIO-hM4Di-EGFP (**Fig 8A and 8B**). LHb-projecting vLGN/IGL neurons were chemogenetically inhibited during bright light treatment (3,000 lux, 2 h/day) (**Fig 8A**). Inhibition of LHb-projecting vLGN/IGL neurons significantly reduced the NREM sleep-reducing effects of bright light treatment in mice that received chronic stress (**Figs 8C, 8D, S8A and S9B**). Next, we injected rAAV2/2-Retro-Cre into the bilateral RMTg and infected RMTg-projecting LHb neurons with AAV2/9-DIO-hM3Dq-EGFP (**Fig 8A and 8E**). RMTg-projecting LHb neurons were chemogenetically activated during bright light treatment (3,000 lux, 2 h/day) (**Fig 8A**). Activation of RMTg-projecting LHb neurons also significantly reduced the NREM sleep-reducing effects of bright light treatment (**Figs 8F, 8G, S8C and S8D**). Finally, we injected AAV2/1-Cre into the bilateral LHb and infected RMTg neurons receiving direct LHb inputs with AAV2/9-DIO-hM3Dq-EGFP (**Fig 8A and 8H**). RMTg postsynaptic neurons were chemogenetically activated during bright light treatment (3,000 lux, 2 h/day) (**Fig 8A**). Activation of RMTg neurons receiving direct LHb inputs significantly impaired the NREM sleep-reducing effects of bright light treatment (**Figs 8I, 8J, S8E and S8F**). Taken together, these findings indicate that activation of the vLGN/IGL-LHb-RMTg pathway is required for the ability of bright light treatment to suppress the abnormal increase in NREM sleep duration induced by chronic stress.

## Discussion

Light exerts a profound effect on a variety of brain functions, including sleep [29,30]. However, the neuronal circuits underlying the effects of light on sleep regulation remain to be elucidated. In this study, we demonstrate that chronic stress promotes NREM sleep by activating the LHb-RMTg pathway, whereas bright light signals transmitted by the vLGN/IGL reduce the NREM sleep-promoting effects of chronic stress by inhibiting neuronal activity in the LHb-RMTg pathway.

Stressful stimuli are powerful modulators of sleep in various species, including humans [1]. In rodents, both acute and chronic stress have been reported to alter sleep patterns [31–34]. Consistently, we found that exposure to 2 weeks of stressful stimuli significantly increased NREM sleep duration and decreased wakefulness time in mice. Given that the LHb is implicated in encoding stressful signals, we postulated that the LHb might be important for the NREM sleep-promoting effects of chronic stress. In support of this possibility, we found that activation of the LHb is both necessary and sufficient for the NREM sleep-promoting effects of chronic stress. It is well established that the LHb can directly innervate several brain regions associated with sleep regulation. It is reasonable for us to speculate that the LHb might mediate the NREM sleep-promoting effects of chronic stress through certain downstream targets. We found that specific activation of RMTg-projecting LHb neurons best mimics the NREM sleep-promoting effects of chronic stress. Our finding that the LHb activates the RMTg through direct projections and that RMTg neurons encode stressful signals suggests that alterations in neuronal activity in the RMTg also regulate the effects of chronic stress on sleep. We found that activation of RMTg neurons receiving direct LHb inputs promoted NREM sleep, whereas inhibition of the RMTg during exposure to stressful stimuli reduced the NREM sleep-promoting effects of chronic stress. These findings strongly suggest that chronic stress can promote NREM sleep by activating the LHb-RMTg pathway, which is also consistent with recent findings that the RMTg plays a prominent role in the regulation of NREM sleep [35]. Interestingly, we found that although long-term activation of DRN-projecting LHb neurons or VTA-projecting LHb neurons did not mimic the effects of chronic stress on sleep states during the nighttime, these manipulations promoted NREM sleep during the daytime. These results suggest that the LHb-DRN/VTA pathways might also contribute to the effects of chronic stress on sleep states, and future studies should be conducted to elucidate their contributions.

We observed that the NREM sleep duration of mice was significantly increased after long-term exposure to chronic stress, and that activation of the LHb-RMTg pathway was needed for the NREM sleep-promoting effects of chronic stress. These results suggest that long-term exposure to chronic stress may influence the intrinsic physiological properties of the LHb-RMTg pathway. Consistent with this view, we found that long-term exposure to chronic stress significantly increased the excitability of RMTg-projecting LHb neurons and RMTg neurons receiving direct LHb inputs (**Fig 7H and 7I**), suggesting that the altered intrinsic physiological properties of the LHb-RMTg pathway may be responsible for the increased NREM sleep resulting from long-term exposure to chronic stress. Although it is well established that chronic stress can increase the excitability of LHb neurons [8,14], the underlying mechanisms remain unclear. The LHb receives excitatory inputs from several brain regions that encode stressful stimuli, including the lateral hypothalamus (LH), the globus pallidus (GPi), and ventral pallidum (VP) [10]. Therefore, the chronic stress-induced increase in the excitability of RMTg-projecting LHb neurons may be due to increased excitatory inputs from these upstream brain regions of the LHb. Additionally, our findings suggest that activation of LHb neurons activates RMTg neurons through a glutamate-mediated mechanism (**Fig 4E**). Thus, the chronic stress-induced increase in the excitability of RMTg postsynaptic neurons observed in our study may be due to the activation of LHb neurons caused by chronic stress.

The vLGN/IGL in rodents is homologous to the pregeniculate nucleus in primates [36–39]. Recent studies conducted in rodents indicate an indispensable role for the vLGN/IGL in mediating the effects of bright light on diverse brain functions, including depression, memory, anxiety, defensive behaviors, and pain [8,40–43]. In this study, we found that vLGN/IGL GABA neurons directly innervated the LHb-RMTg pathway. Given that the vLGN/IGL encodes bright light signals [8,40–43] and that activation of the LHb-RMTg pathway is needed for the NREM sleep-promoting effects of chronic stress, it is plausible that bright light signals transmitted by the vLGN/IGL regulate neuronal activity in the LHb-RMTg pathway and influence the effects of chronic stress on sleep. This hypothesis is supported by several lines of evidence. First, we found that bright light treatment reduced the NREM sleep-promoting effects of chronic stress and simultaneously decreased the excitability of the LHb-RMTg pathway. In addition, we demonstrated that the effect of bright light treatment on sleep was abolished by inhibition of LHb-projecting vLGN/IGL neurons or the LHb-RMTg pathway.

In our previous study, we demonstrated that M4-type melanopsin-expressing retinal ganglion cells (M4-mRGCs) directly project to the vLGN/IGL and inhibit the LHb through these projections [8]. These findings suggest that bright light stimulation or activation of the vLGN/IGL could lead to inhibition of neuronal activity in the LHb. Our results further support this prediction as both chemogenetic activation of the vLGN/IGL and exposure to bright light (3,000 lux) reduced the excitatory effects of stressful stimuli on the LHb [8]. Additionally, we observed that short-term exposure to bright light stimulation (3,000 lux, 2 h) did not increase c-Fos expression in the LHb [8], which is consistent with prior research indicating that specific activation of mRGCs does not significantly enhance c-Fos expression in the LHb [44]. These results strongly indicate that bright light predominantly exerts an inhibitory effect on neuronal activity in the LHb. Notably, a previous electrophysiological study reported the presence of light-induced increases in the activity of LHb neurons [45]. We think there are 2 reasons for this phenomenon. Firstly, studies have shown that mRGCs, mainly M1 type, can directly project to the perihabenular nucleus (pHb) [46,47], which is situated close to the LHb. Therefore, some of the light-sensitive neurons recorded by the electrode may be located in the pHb. Secondly, it is also plausible that light may activate a small number of LHb neurons by engaging certain neuromodulatory systems in the brain [10], suggesting that the effects of light on LHb neurons are heterogeneous. Future studies will be necessary to establish the function of LHb neurons that may be activated by light stimulation.

Previous studies have shown that a subset of mRGCs in mice, mainly M1, directly innervates the pHb, which is a previously unrecognized structure of the dorsal thalamus. Unlike the LHb of the epithalamus, pHb can be activated by dark-light transitions [46] or light exposure at night [47], but not by exposure to stressful stimuli. The authors demonstrated that chronic activation of the pHb through exposure to an ultradian light-dark cycle (T7 cycle; alternating 3.5-h periods of light and darkness) [46] or light at night [47] increased depressive-like behaviors. In contrast, our previous study found that bright light treatment during the day decreased depressive-like behaviors induced by chronic stress through activation of the retina-vLGN/IGL-LHb pathway [8]. However, the question arises as to why plenty of light during the day, which should activate the mRGC-pHb pathway and increase depressive-like behaviors, has an antidepressant effect. An and colleagues found that the excitability of pHb neurons is higher at night than during the day, and they tend to transmit nighttime light information, indicating that the pHb can act as a valve controlled by the circadian rhythm [47]. Specifically, it mediates the regulation of negative mood by nocturnal light messages. Combining this result with our previous finding that light treatment during the day decreased depressive-like behaviors induced by exposure to chronic stress through the inhibition of LHb [8], it appears that even if both the retina-vLGN/IGL-LHb pathway and mRGC-pHb pathway were activated during light treatment, the depression-inducing effects caused by the activation of the mRGC-pHb pathway were negated by the antidepressant effects induced by the activation of the retina-vLGN/IGL-LHb pathway.

The response to environmental stressors comprises profound physiological changes that ensure survival and restore homeostasis [48,49]. The increase in NREM sleep duration after exposure to chronic stress observed in this study might reflect a mechanism for alleviating the malign effects of chronic stress [34]. Interestingly, we demonstrated that bright light treatment decreased the depression-inducing effects of chronic stress [8] and reduced the NREM sleep-promoting effects of chronic stress. This suggests that bright light treatment can increase resistance to stress, which may lead to a reduction in the body’s need to restore homeostasis through excessive NREM sleep in response to chronic stress [49].

Taken together, our findings provide direct evidence that the LHb-RMTg pathway plays a pivotal role in mediating the NREM sleep-promoting effect of chronic stress and that bright light treatment can decrease the NREM sleep-promoting effect of chronic stress through a di-synaptic visual circuit consisting of the vLGN/IGL, LHb and RMTg (**S9 Fig**). These results may further deepen our understanding of the structure and function of the non-image-forming system and provide a theoretical basis for the application of bright light treatment for the alleviation of stress-related disorders.

## Materials and methods

### Ethics statement

All experiments were approved by the Jinan University Institutional Animal Care and Use Committee. The approval number of the protocol is 20220804–14. We also abide by the provisions of the Biosafety Law of the People’s Republic of China, the Regulations on the Administration of Experimental Animals, the National Standards for Experimental Animals (GB14925-2010), the Guidelines for Ethical Review of the Welfare of Experimental Animals (GBT 35892–2018), and the relevant rules and regulations formulated by Jinan University.

### Animals

Adult (6 to 8 weeks old) male C57BL/6 mice were used in this study. The animals were housed in a 12 h:12 h light-dark cycle [light on at 7 AM, this time point is defined as Zeitgeber time (ZT) 0] with food and water provided ad libitum. The animals were randomly allocated to experimental and control groups. Experimenters were blind to the experimental group, and the order of testing was counterbalanced during behavioral experiments.

### Surgery and intracranial injection

The mice were anesthetized (Avertin, 13 μl/g, i.p.) and placed in a stereotaxic instrument (RWD, Shenzhen, China). Erythromycin eye ointment was applied to prevent corneal drying and a heat pad (RWD, Shenzhen, China) was used to hold body temperature at 37°C. A small craniotomy hole was made using a dental drill (OmniDrill35, WPI, Sarasota, Florida, United States of America), and injections were performed via a micropipette connected to a Nanoliter Injector (NANOLITER 2010, WPI, Sarasota, Florida, USA) and its controller (Micro4, WPI, Sarasota, Florida, USA) at a slow flow rate of 0.1 μl/min to avoid potential damage to local brain tissue.

To specifically infect LHb neurons with hM3Dq-EGFP or hM4Di-EGFP or EGFP, AAV2/9-hSyn-hM3Dq-EGFP or AAV2/9-hSyn-hM4Di-EGFP or AAV2/9-hSyn-EGFP was injected into the LHb of C57BL/6 mice (virus titers: 3.5 × 10^12^ GC/ml, 0.2 μl/injection; *AP*: −1.6 mm; *ML*: ± 0.25 mm; *DV*: −3.05 mm), respectively.

To specifically infect RMTg-projecting LHb neurons with ChR2-mCherry or mCherry or hM3Dq-EGFP or hM4Di-EGFP or EGFP or eYFP or GCaMP7s, rAAV2/2-Retro-Cre was injected into the RMTg of C57BL/6 mice (virus titers: 3 × 10^12^ GC/ml; 0.1 μl/injection; *AP*: −4.3 mm; *ML*: ±0.35 mm; *DV*: −4.25 mm), AAV2/9-DIO-ChR2-mCherry or AAV2/9-DIO-mCherry or AAV2/9-DIO-hM3Dq-EGFP or AAV2/9-DIO-hM4Di-EGFP or AAV2/9-DIO-EGFP or AAV2/9-DIO-eYFP or AAV2/9-DIO-GCaMP7s was injected into the LHb (virus titers: 3.5 × 10^12^ GC/ml, 0.2 μl/injection; *AP*: −1.6 mm; *ML*: ± 0.25 mm; *DV*: −3.05 mm), respectively.

To specifically infect DRN-projecting LHb neurons with hM3Dq-EGFP or EGFP, rAAV2/2-Retro-Cre was injected into the DRN of C57BL/6 mice (virus titers: 3 × 10^12^ GC/ml; 0.2 ml/injection; *AP*: −5.5 mm; *DV*: −2.7 mm, with a 15°aangle), AAV2/9-DIO-hM3Dq-EGFP or AAV2/9-DIO-EGFP was injected into the LHb (virus titers: 3 × 10^12^ GC/ml; 0.1 μl/injection), respectively.

To specifically infect VTA-projecting LHb neurons with hM3Dq-EGFP or EGFP, rAAV2/2-Retro-Cre was injected into the VTA of C57BL/6 mice (virus titers: 3 × 10^12^ GC/ml; 0.2 ml/injection; *AP*: −3.2; *ML*: ± 0.5 mm; *DV*: −4.25 mm), AAV2/9-DIO-hM3Dq-EGFP or AAV2/9-DIO-EGFP was injected into the LHb (virus titers: 3 × 10^12^ GC/ml; 0.1 μl/injection), respectively.

To specifically infect postsynaptic RMTg neurons with EGFP or hM3Dq-EGFP or hM4Di-EGFP or GCaMP7s, AAV2/1-Cre (virus titers: 1.5 × 10^13^ GC/ml; 0.1 μl/injection) was injected into the LHb, AAV2/9-DIO-EGFP or AAV2/9-DIO-hM3Dq-EGFP or AAV2/9-DIO-hM4Di-EGFP or or AAV2/9-DIO-GCaMP7s was injected into the RMTg (0.15 μl/injection), respectively.

For di-synaptic tracing vLGN/IGL-LHb-RMTg pathway, 0.15 μl Helper virus (rAAV2/9-hSyn-EGFP-2a-TVA-2a-RVG-WPRE-pA) (virus titers: 2 × 10^8^ GC/ml) was injected into the LHb. Twenty-one days later, 0.1 μl of SAD-DG-DsRed (EnvA) (RV-DsRed, virus titers: 2 × 10^8^ GC/ml) was injected into RMTg.

Following injection, the micropipette was left in place for approximately 5 min and then extracted slowly (approximately 1 min to completely move the micropipette from the injection site to the surface of the brain) to minimize virus leakage in the track. Finally, the wound was sutured, antibiotics (bacitracin and neomycin) were applied to the surgical wound and ketoprofen (5 mg/kg) was injected subcutaneously; the animals were allowed to recover from anesthesia under a heat lamp.

### Verification of the location of the RMTg

Previous studies have demonstrated that cocaine can selectively activate the RMTg after its rewarding effects have subsided [50–53]. In this study, we further assessed the location of the RMTg based on cocaine-induced c-Fos expression. Briefly, mice received an i.p. injection of cocaine (10 mg/kg). One hour later, the animals were anesthetized (13 μl/g avertin, i.p.) before receiving an intracardial infusion of 0.9% saline, followed by 4% paraformaldehyde in PBS. The brains were carefully removed thereafter. To label c-Fos^+^ cells, cryostat sections were blocked for 1 h and then incubated with a primary anti c-Fos antibody (rabbit, 1:500, 2250, Cell Signaling Technology) for 36 h at 4°C. The sections were subsequently incubated with a secondary antibody, DyLight 594-conjugated goat anti-rabbit IgG (DI-1 594, Vector Laboratories) at a dilution of 1:400 for 6 h at room temperature. Finally, all sections were washed in 0.1 M PBS and mounted with an antifade aqueous mounting medium containing DAPI (EMS, Hatfield, Pennsylvania, USA). Consistent with previous findings, we observed an increase in c-Fos expression in the RMTg, but not the VTA, 1 h after cocaine exposure (**S10A Fig**).

### Injection site verification

After transcardial perfusion with 0.9% saline followed by 4% paraformaldehyde in 0.1 M PBS, the brain was removed and post-fixed with 4% paraformaldehyde overnight at 4°C, and then transferred into 30% sucrose until sectioning with a cryostat (CM1900, Leica Microsystems, Bannockburn, Illinois, USA). A series of 40 μm sections were collected for verification of injection sites. To confirm the injection sites of viruses that encoded a fluorescent protein (e.g., AAV2/9-DIO-hM3Dq-EGFP and AAV2/9-DIO-GCaMP7s) (**S10B and S10C Fig**), coronal brain sections were examined under a fluorescence microscope (Zeiss, Axioimager Z2 microscope). Only mice with verified fluorescent protein expression were used for analysis. Mice with virus injections that missed the target area, fluorescent protein expression that was too low in the targeted area or extensively extended beyond the targeted area were excluded from the study. To visualize the injection sites of viruses that did not encode fluorescent protein (i.e., rAAV2/2-Retro-Cre and AAV2/1-Cre), Alexa Fluor 647-conjugated cholera toxin subunit B (CTB-647, 0.05 μl/injection) was injected into the targeted regions along with the viruses. The location of the virus injection site was visualized with CTB-647 (**S10D Fig**). Only mice with verified injection sites were used for analysis.

### EEG/EMG recordings and analysis of sleep-wake states

We implanted electrodes on day 7 of daily exposure to chronic stress or i.p. injection of CNO. The animals continued to receive daily exposure to chronic stress or i.p. injection of CNO for 7 days after electrode implantation. Two days before the end of the 14-day period of daily exposure to chronic stress or i.p. injection of CNO, the animals were transferred to the recording room and habituated to the recording cables and conditions for 2 days. Following this habituation period, EEG/EMG recordings (8400-K1, PINNACLE, USA) were performed on all animals for 24 h.

Original data were converted into European data format (edf). Sleep states were scored with sleep analysis software (SleepSign, Kissei Comtec). Cortical EEG and EMG signals were amplified, filtered (EEG, 0.5 to 30 Hz; EMG, 20 to 200 Hz), digitized at a sampling rate of 128 Hz, and recorded using Pinancle’s recoding equipment (8400-K1, PINNACLE, USA). When complete, polygraphic recordings were automatically scored offline by 4-s epochs as wake, NREM sleep, and REM sleep using SleepSign according to standard criteria. EEG power in the ranges from 0.5 to 4 Hz and from 5 to 8 Hz were defined as delta and theta power, respectively. The general criteria for classification of the different states were: wake = with locomotor activity, high EMG, and intermediate theta/delta wave ratio; NREM sleep = no locomotor activity, low EMG, and predominated with delta wave; REM sleep = no locomotor activity, low EMG, and predominated with theta wave. All classifications of states automatically assigned by SleepSign were examined visually and corrected manually. The total durations and episodes numbers of wake, NREM and REM were exported as a text file. Spectrograms were generated using Sirenia Sleep Pro software (PINNACLE, USA).

### Fiber photometry

A fiber photometry system was used for recording Ca^2+^ signals from RMTg-projecting LHb neurons or RMTg neurons receiving direct LHb inputs. Briefly, 1 week after viruses injection, an optical fiber [O.D. = 230 mm, numerical aperture (NA) = 0.37] was placed in a ceramic ferrule and inserted towards the LHb (coordinates: *AP*: −1.6 mm; *ML*: ± 0.35 mm; *DV*: −2.6 mm) or RMTg (coordinates: *AP*: −4.3 mm; *ML*: ± 0.35 mm; *DV*: −4.2 mm) through the craniotomy. The ceramic ferrule was supported with a skull penetrating M1 screw and dental acrylic. Mice were individually housed and allowed to recover for at least 1 week. To record fluorescence signals, GCaMP7s fluorescence was detected through the optic fiber using a fiber photometry system (Inper, China). Calcium-dependent fluorescence signals were obtained by stimulating LHb or RMTg neurons expressing GCaMP7s with a 470 nm LED (40 μW at fiber tip) while calcium-independent signals were obtained by stimulating these cells with a 410 nm LED (20 μW at fiber tip), which was further used to correct for movement artifacts. The 410 nm signal was scaled using least-squares regression to minimize the difference between the 410 and 470 signal. The fitted 410 nm signal was then subtracted from the 470 nm signal to obtain the movement and bleaching-corrected signal. The values of fluorescence change (ΔF/F) by calculating (F−F0)/F0, where F0 was the averaged baseline fluorescence signal recorded before stressful stimuli.

For the foot shock stimulation, foot shocks (1 mA, 500 ms) were delivered with inter-trial intervals of 15 s. Shock delivery onset was used as the trigger event for data alignment. For the air puff stimulation, the body of the mouse was inserted into an acrylic “body tube,” with the mouse’s head extending out. A brief air puff was delivered to the face of the mouse by pressing a hand-pump air compressor (13 × 5 cm) attached to a PVC tube with its opening positioned approximately 5 cm away from the mouse’s nostril. One press of the hand pump generates a ~2-s-long gentle air puff. Air puffs were delivered with inter-trial intervals of approximately 15 s. Onset of air puff delivery was used as the trigger event for data alignment.

### Physiological recording from brain slices

For brain slice preparation, the mice were deeply anesthetized with isoflurane, and coronal sections (250 μm thick) containing the LHb or RMTg were cut using a vibratome (VT1200S; Leica Microsystems) in ice-cold artificial cerebrospinal fluid (ACSF, in mM: 119 NaCl; 2.5 KCl, 1 NaH_2_PO_4_, 11 glucose, 26.2 NaHCo_3_, 2.5 CaCl_2_, 1.3 MgCl_2_, and 290 mOsm, at pH 7.4). The brain slices were recovered for approximately 1 h at room temperature in ACSF. After recovery, the slices were placed in the recording chamber and continuously perfused with ACSF.

Evoked postsynaptic currents were elicited by 2 ms blue light stimulation of axonal terminals of RMTg-projecting LHb neurons infected with ChR2-mCherry. Blue-light-evoked EPSCs and IPSCs were recorded when the membrane potential was held at −70 mV and 0 mV, respectively. To test whether the recorded EPSCs were mediated by the AMPA/kainate receptor, 10 μM NBQX was added to ACSF. To test whether the postsynaptic currents recorded in RMTg neurons were elicited by direct synaptic connections, 1 μM tetrodotoxin (TTX) and 100 μM 4-aminopyridine (4-AP) were added to ACSF. The recorded cells were intracellularly filled with biocytin for morphological evaluation.

To measure the excitability of RMTg-projecting LHb neurons or RMTg neurons receiving direct LHb inputs, electrodes were filled with K^+^-based peptide solution (in mM: 130 KMeSO_4_, 10 KCl, 10 Na_2_-phosphocreatine, 4 MgATP, 0.3 Na_3_GTP, 10 HEPES, 290 mOsm, adjusted to 7.4 with KOH). Current steps were applied (for RMTg-projecting LHb neurons: 10 pA injection current per step, duration: 1 s; for RMTg neurons receiving direct LHb inputs: 20 pA injection current per step, duration: 1 s) from a membrane potential of −70 mV.

To measure the function of chemogenetic viruses, neurons expressing hM3Dq-EGFP or hM4Di-EGFP in the LHb or RMTg were recorded. For chemogenetic activation, hM3Dq-EGFP-labeled neurons were recorded in the current-clamp model. After 80 s of baseline recording, 10 μM CNO was washed into ACSF for 100 s, and the neurons were recorded for 4 min in total. For chemogenetic inhibition, hM4Di-EGFP-labeled neurons were injected with a 100 pA current, and the number of activated action potentials was calculated as the baseline. Then, 10 μM CNO was added to ACSF for 10 min, and the action potentials activated by 100 pA current injection were recorded. Finally, the CNO was washed out, and the activated action potentials were recorded.

All recordings were performed using a Multiclamp 700B amplifier (Molecular Devices). Traces were low-pass-filtered at 2 kHz and digitized at 10 kHz. For light stimulation, light pulses were delivered through digital commands from the Digidata 1550A and Digital stimulator (PG4000a, Cygnus Technology). The pipette resistance ranged from 4 to 6 MΩ. When stable whole-cell recordings were achieved with an access resistance below 25 MΩ, basic electrophysiological properties were recorded. Offline data analysis was performed using Clampfit 10.0 software (Molecular Devices).

### Immunocytochemistry

All animals were anesthetized (Avertin, 13 ml/g, intraperitoneally) and perfused intracardially with 0.9% saline followed by 4% paraformaldehyde in phosphate-buffered saline (PBS). Brains were removed. For CaMKIIα labeling, 40 μm cryostat sections containing the LHb were placed in blocking solution for 1 h before incubation in primary antibody against CaMKIIα (rabbit, 1:500; ab5683, Abcam) (36 h at 4°C). Sections were then incubated with corresponding secondary antibody at a dilution of 1:400 for 6 h at room temperature: (Dylight 594) goat-anti-rabbit IgG (DI-1649, Vector Laboratories). For GABA labeling, 40 μm cryostat sections containing the RMTg were placed in blocking solution for 1 h before incubation in primary antibody against GABA (rabbit, 1:500; A2052, sigma) (36 h at 4°C). Sections were then incubated with corresponding secondary antibody at a dilution of 1:400 for 6 h at room temperature: (Dylight 594) goat-anti-rabbit IgG (DI-1649, Vector Laboratories).

### Image analysis

Brain sections were imaged with a Zeiss 700 confocal microscope with 5× or 20× objectives, or a 40× oil immersion objective. For three-dimensional reconstruction of injected or virus labeled cells, optical sections were collected at 0.2 μm intervals. Using Image J and Photoshop CS5 (Adobe Corp., San Jose, California, USA), each stack of optical sections was montaged and projected to a 0° X-Y plane and a 90° Y-Z plane to obtain a three-dimensional reconstruction of the cell. Contrast and brightness were adjusted, and the red-green images had been converted to magenta-green.

### Behavioral paradigms

Behavioral tests were performed during the light phase (ZT 6 to ZT 9) unless otherwise specified. Operators were blinded to the experimental group during scoring.

#### Exposure to chronic stress

To evaluate the influence of chronic stress on sleep/wake states and depressive-like behaviors, we used the chronic stress protocol described in our previous study [8] with slight modifications. Briefly, mice were exposed to a series of stressful stimuli (foot shocks, air puffs, and physical restraint) from ZT 6 to ZT 9 each day for 2 weeks. Foot shocks (20 times/day, 1 mA, 500 ms) were randomly delivered with inter-trial intervals of 15, 20, or 30 s to mice placed in an acrylic box (25 cm × 25 cm × 40 cm) equipped with a metal grid floor. For air puff stimulation, mice were placed in their home cages and received random air puffs (30 times/day) to the face with inter-trial intervals of 10 to 15 s. Physical restraint involved placing mice in a plastic restrainer for 1 h per day. The interval between the 3 stressors was 30 min.

#### Light treatment

The animals in both the control and light treatment groups were kept in their home cages which were placed on different layer of a custom-designed light cabinet for 2 weeks, where all animals were housed at room temperature with ad libitum access to food and water. Cool LED lights (UV-free) with adjustable brightness was installed at the top of each floor of the cabinet so that the brightness of each floor of the cabinet could be adjusted manually (the light intensity was determined by averaging the measurements from the top and the 4 sides of the cage). The animals in the control group were housed under a ZT 0 to ZT 24 12 h:12 h light/dark cycle (approximately 200 lux white ambient illumination). The animals in the experimental group were also housed under a ZT 0 to ZT 24 12 h:12 h light/dark cycle (approximately 200 lux white ambient illumination) except for during light treatment (approximately 3,000 lux white ambient illumination between ZT 1 and ZT 3). Following housing in the light cabinet, all animals underwent behavioral tests as detailed below.

#### Sucrose preference test (SPT)

Mice were tested for preference for a 2% sucrose solution (Sucrose, Sigma-Aldrich) using a two-bottle choice procedure. Each animal was housed individually during the 2-day test period. Animals were given 2 bottles, one of sucrose and one of tap water. Every 24 h, the amounts of sucrose and water consumed were recorded. To prevent potential location preference for drinking, the positions of the bottles were changed every 24 h. Food and water were available ad libitum prior to the SPT. The preference for the sucrose solution was determined as the percentage of sucrose solution ingested relative to the total intake.

#### Forced swimming test (FST)

Mice were placed in a cylinder of water (temperature of 23 to 25°C; 20 cm in diameter, 27 cm in height for mice) for 6 min. The depth of water was set to prevent animals from touching the bottom with their hind limbs. Animal behavior was video-tracked from the side. The time each animal spent immobile during the test was counted online by 2 independent observers in a blinded manner. Immobility was defined as no active movement except that needed to keep the animal from drowning.

#### Tail suspension test (TST)

Each mouse was suspended to a rod by its tail with an adhesive tape at 55 cm above the surface. Total immobility duration was measured during 6 min. The test was videotaped and immobility time was analyzed by an independent observer.

#### Open field test (OFT)

Motor activity was measured in a white Plexiglas arena (50 cm length × 50 cm width × 40 cm height). Briefly, the mice were placed in the center of a plastic box with dim light (15 lux) and were allowed to explore the arena for 10 min. All animal activity was recorded with an infrared camera placed above the box. Locomotion and time spent in the center during the 10 min of exploration was measured (Ethovision XT software). The box was wiped clean with a paper towel soaked in 50% ethanol and dried thoroughly after each test session.

#### Wheel-running test (WRT)

Mice were individually housed in cages equipped with a running wheel (110 mm diameter). Cool LED lights (UV-free) with adjustable brightness were installed at the top of each cage. All animals were housed at room temperature with ad libitum access to food and water. All animals were housed under a ZT 0 to ZT24 12 h:12 h light/dark cycle (200 lux white ambient illumination). The number of wheel revolutions was counted by a custom-made drive based on Ar Control [54]. The activity onset and locomotor activity in 5 min time bins during a period of 8 days were analyzed using MATLAB and GraphPad software.

### Chemogenetic manipulation

The designer drug CNO (1 mg/kg, i.p.; C0832, Sigma-Aldrich, St Louis, Missouri, USA) was administrated 30 min before exposure to stressful stimuli (ZT 5.5, **Figs 2C, 6A and S7G**) or bright light treatment (ZT 0.5, **Fig 8A**). For **Figs 2H, 3A, 5D**, **S1A** and **S7C**, CNO (1 mg/kg, i.p.) was administrated between ZT 3 to ZT 3.5.

### Quantification of neurons infected with different viruses

To quantify percentage of RV-DsRed labeled vLGN/ IGL neurons co-labeled with GABA (**Fig 7C**), 4 C57BL/6 mice received LHb injection of helper virus and RV-DsRed were used. In each mouse, the number of RV-DsRed-labeled vLGN/IGL neurons and RV-DsRed/GABA double labeled vLGN/IGL neurons were counted in 4 serial brain sections (40 μm/section) across the vLGN/IGL. The percentage of RV-DsRed/GABA double labeled neurons was calculated as a percentage of the total number of RV-DsRed/GABA double labeled vLGN/IGL neurons counted in 4 mice from the total number of RV-DsRed-labeled neurons counted in 4 mice.

To quantify percentage of RMTg-projecting LHb neurons co-labeled with CaMKIIα (**S4A Fig**), 4 C57BL/6 mice received RMTg injection of rAAV2/2-Retro-Cre and LHb injection of AAV2/9-DIO-eYFP were used. In each mouse, the number of eYFP-labeled LHb neurons and eYFP/CaMKIIα double labeled LHb neurons were counted in 4 serial brain sections (40 μm/section) across the LHb. The percentage of eYFP/CaMKIIα double labeled neurons was calculated as a percentage of the total number of eYFP/CaMKIIα double labeled LHb neurons counted in 4 mice from the total number of eYFP neurons counted in 4 mice.

To quantify percentage of RMTg neurons receiving direct LHb inputs co-labeled with GABA (**S4B Fig**), 4 C57BL/6 mice received LHb injection of AAV2/1-Cre and RMTg injection of AAV2/9-DIO-eYFP were used. In each mouse, the number of eYFP-labeled RMTg neurons and eYFP/GABA double labeled LHb neurons were counted in 4 serial brain sections (40 μm/section) across the RMTg. The percentage of eYFP/GABA double labeled neurons was calculated as a percentage of the total number of eYFP/GABA double labeled RMTg neurons counted in 4 mice from the total number of eYFP neurons counted in 4 mice.

### Statistics

All statistics were calculated using GraphPad Prism 7 software. Data analysis was done by experimenters blind to experimental conditions. Statistical details including the definitions and exact value of *n* (e.g., number of animals), *p* values, and the types of the statistical tests can be found in the Figures and Figure legends. One-way ANOVA and then *Sidak’s* multiple comparisons test was used to quantify the performance of the SPT, FST, TST, OFT tests, the amplitude of the IPSC and EPSC, and number of action potentials activated by current injection. For all figures, dot plots include horizontal line representing mean. Statistical significance was set at *P* < 0.05.

## Supporting information

S1 Fig The effects of inhibition of the LHb on sleep/wake states. **(A)** Schematic of the experimental design. **(B)** Representative EEG spectrograms, EEG and EMG traces (recorded from ZT 1 to ZT 2) of mice in different experimental groups. All animals received i.p. injection of CNO (1 mg/kg). EGFP–CNO: mice that received LHb injection of AAV2/9–hSyn–EGFP; hM4Di–CNO: mice that received LHb injection of AAV2/9–hSyn–hM4Di–EGFP. **(C)** Left: time course changes of NREM and REM sleep and wakefulness of mice in EGFP–CNO and hM4Di–CNO groups (*n* = 6 animals/group). Middle: total sleep–wake amounts during the whole day (24 h) of mice in in EGFP–CNO and hM4Di–CNO groups. Right: total sleep–wake amounts during light phase (ZT 0 –ZT 12) and dark phase (ZT 12 –ZT 24) of mice in in EGFP–CNO and hM4Di–CNO groups. **(D)** Number of transitions between different pair of brain states during the light phase (ZT 0 –ZT 12), dark phase (ZT 12 –ZT 24), and the whole day (24 h) of mice in EGFP–CNO and hM4Di–CNO groups (*n* = 6 animals/group). W–N: Wake to NREM; N–W: NREM to Wake; N–R: NREM to REM. **(E)** Left: time course changes of REM sleep and wakefulness of mice in different experimental groups (*n* = 6 animals/group). All animals received exposure to chronic stress stimuli (CS) and i.p. injection of CNO (1 mg/kg). CS+EGFP–CNO: mice that received LHb injection of AAV2/9–hSyn–EGFP. CS+hM4Di–CNO: mice that received LHb injection of AAV2/9–hSyn–hM4Di–EGFP. Middle: total REM sleep and wakefulness amounts during the whole day (24 h) of mice in CS+EGFP–CNO and CS+hM4Di–CNO groups (*n* = 6 animals/group). Right: REM sleep and wakefulness amounts during light phase (ZT 0 –ZT 12) and dark phase (ZT 12 –ZT 24) of mice in CS+EGFP–CNO and CS+hM4Di–CNO groups (*n* = 6 animals/group). **(F)** Number of transitions between different pair of brain states during the light phase (ZT 0 –ZT 12) and dark phase (ZT 12 –ZT 24) of animals in CS+EGFP–CNO and CS+hM4Di–CNO groups (*n* = 6 animals/group). W–N: Wake to NREM; N–W: NREM to Wake; N–R: NREM to REM. For all figures: one–way ANOVA with *Sidak’s* multiple comparisons test, *, *P* < 0.05; **, *P* < 0.001; ***, *P* < 0.0001; ns = no significant difference. Error bars indicate the SEM. Underlying data can be found in S1 Data.
(TIF)

S2 Fig Effects of activation of the LHb on depressive–like behaviors and sleep/wake states. **(A)** Depressive–like behaviors in different experimental groups (*n* = 7 animals/group). All animals received i.p. injection of CNO (1 mg/kg). EGFP–CNO: mice that received LHb injection of AAV2/9–hSyn–EGFP; hM3Dq–CNO: mice that received LHb injection of AAV2/9–hSyn–hM3Dq–EGFP. (**B**) Left: time course changes of REM sleep and wakefulness of mice in in different experimental groups. All animals received i.p. injection of CNO (1 mg/kg). EGFP–CNO (*n* = 6 animals): mice that received LHb injection of AAV2/9–hSyn–EGFP. hM3Dq–CNO (*n* = 7 animals): mice that received LHb injection of AAV2/9–hSyn–hM3Dq–EGFP; Middle: total REM sleep and wakefulness amounts during the whole day (24 h) of mice in EGFP–CNO (*n* = 6 animals) and hM3Dq–CNO (*n* = 7 animals) groups. Right: REM sleep and wakefulness amounts during light phase (ZT 0 –ZT 12) and dark phase (ZT 12 –ZT 24) of mice in EGFP–CNO (*n* = 6 animals) and hM3Dq–CNO (*n* = 7 animals) groups. **(C)** Number of transitions between different pair of brain states during the light phase (ZT 0 –ZT 12) and dark phase (ZT 12 –ZT 24) of mice in EGFP–CNO (*n* = 6 animals) and hM3Dq–CNO (*n* = 7 animals) groups. For all figures: one–way ANOVA with *Sidak’s* multiple comparisons test, *, *P* < 0.05; **, *P* < 0.001; ***, *P* < 0.0001; ns = no significant difference. Error bars indicate the SEM. Underlying data can be found in S1 Data.
(TIF)

S3 Fig Activation of RMTg–projecting LHb neurons mimics the effects of chronic stress on sleep/wake states. **(A, C, E)** Left: time course changes of REM sleep and wakefulness of mice in different experimental groups. All mice received i.p. injection of CNO (1 mg/kg). EGFP–CNO (*n* = 6 animals/group): mice that received DRN (**A**) or VTA (**C**) or RMTg (**E**) injection of rAAV2/2–Retro–Cre and LHb injection of AAV2/9–DIO–EGFP; hM3Dq–CNO: mice that received DRN (**A**, *n* = 6 animals) or VTA (**C**, *n* = 6 animals) or RMTg (**E**, *n* = 7 animals) injection of rAAV2/2–Retro–Cre and LHb injection of AAV2/9–DIO–hM3Dq–EGFP. Right: REM sleep and wakefulness amounts during the whole day (24 h), light phase (ZT 0 –ZT 12) and dark phase (ZT 12 –ZT 24) of mice EGFP–CNO and hM3Dq–CNO groups. **(B, D, F)** Number of transitions between different pair of brain states during the light phase (ZT 0 –ZT 12) and dark phase (ZT 12 –ZT 24) of mice in EGFP–CNO and hM3Dq–CNO groups. W–N: Wake to NREM; N–W: NREM to Wake; N–R: NREM to REM. For all figures: one–way ANOVA test, *, *P* < 0.05; **, *P* < 0.01; ***, *P* < 0.0001; ns = no significant difference. Error bars indicate the SEM. Underlying data can be found in S1 Data.
(TIF)

S4 Fig LHb CaMKIIα neuron synapse onto RMTg GABA neurons. **(A)** Left: scheme for specific labeling of RMTg–projecting LHb neurons with eYFP. Middle: representative images of the LHb illustrating eYFP–expressing LHb neurons co–labeled with CaMKIIα. Right: pie chart indicates percentage of eYFP–expressing LHb neurons co–labeled with CaMKIIα. **(B)** Left: scheme for specific labeling of RMTg neurons receiving direct LHb inputs with eYFP. Middle: representative images of the RMTg illustrating eYFP–expressing RMTg neurons co–labeled with GABA. Right: pie chart indicates percentage of eYFP–expressing RMTg neurons co–labeled with GABA. Scale bars: 200 μm (A–left, B–left); 50 μm (A–right, B–right). Underlying data can be found in S1 Data.
(TIF)

S5 Fig Activation of the RMTg neurons receiving direct LHb inputs decreases wakefulness. **(A)** Left: time course changes of REM sleep and wakefulness of mice in different experimental groups. All mice received LHb injection of AAV2/1–Cre and i.p. injection of CNO (1 mg/kg). EGFP–CNO (*n* = 6 animals): mice that received RMTg injection of AAV2/9–DIO–EGFP. hM3Dq–CNO (*n* = 7 animals): mice that received RMTg injection of AAV2/9–DIO–hM3Dq–EGFP. Middle: total REM sleep and wakefulness amounts during the whole day (24 h) of mice in n EGFP–CNO (*n* = 6 animals) and hM3Dq–CNO groups (*n* = 7 animals). Right: REM sleep and wakefulness amounts during light phase (ZT 0 –ZT 12) and dark phase (ZT 12 –ZT 24) of mice in n EGFP–CNO (*n* = 6 animals) and hM3Dq–CNO (*n* = 7 animals) groups. **(B)** Number of transitions between different pair of brain states during the light phase (ZT 0 –ZT 12) and dark phase (ZT 12 –ZT 24) of mice in EGFP–CNO (*n* = 6 animals) and hM3Dq–CNO (*n* = 7 animals) groups. W–N: Wake to NREM; N–W: NREM to Wake; N–R: NREM to REM. For all figures: one–way ANOVA test, *, *P* < 0.05; **, *P* < 0.01; ***, *P* < 0.0001; ns = no significant difference. Error bars indicate the SEM. Underlying data can be found in S1 Data.
(TIF)

S6 Fig Activation of the LHb–RMTg pathway is required for the effects of chronic stress on wakefulness. **(A)** Left: time course changes of REM sleep and wakefulness of mice in different experimental groups. All animals received exposure to chronic stress stimuli (CS), RMTg injection of rAAV2/2–Retro–Cre and i.p injection of CNO (1 mg/kg). CS+EGFP–CNO (*n* = 6 animals): mice that received LHb injection of AAV2/9–DIO–EGFP. CS+hM4Di–CNO (*n* = 7 animals): mice that received LHb injection of AAV2/9–DIO–hM4Di–EGFP. Middle: total REM sleep and wakefulness amounts during the whole day (24 h) of mice in CS+EGFP–CNO (*n* = 6 animals) and CS+hM4Di–CNO (*n* = 7 animals) groups. Right: REM sleep and wakefulness amounts during the whole day (24 h), light phase (ZT 0 –ZT 12) and dark phase (ZT 12 –ZT 24) of mice in CS+EGFP–CNO (*n* = 6 animals) and CS+hM4Di–CNO (*n* = 7 animals) groups. **(B)** Number of transitions between different pair of brain states during the light phase (ZT 0 –ZT 12) and dark phase (ZT 12 –ZT 24) of mice in CS+EGFP–CNO (*n* = 6 animals) and CS+hM4Di–CNO (*n* = 7 animals) groups. W–N: Wake to NREM; N–W: NREM to Wake; N–R: NREM to REM. **(C)** Left: time course changes of REM sleep and wakefulness of mice in different experimental groups (*n* = 7 animals/group). All animals received exposure to CS, LHb injection of AAV2/1–Cre and i.p. injection of CNO (1 mg/kg). CS+EGFP–CNO: mice that received RMTg injection of AAV2/9–DIO–EGFP. CS+hM4Di–CNO: mice that received RMTg injection of AAV2/9–DIO–hM4Di–EGFP. Middle: total REM sleep and wakefulness amounts during the whole day (24 h) of mice in CS+EGFP–CNO and CS+hM4Di–CNO groups (*n* = 7 animals/group). Right: REM sleep and wakefulness amounts during the whole day (24 h), light phase (ZT 0 –ZT 12) and dark phase (ZT 12 –ZT 24) of mice in CS+EGFP–CNO and CS+hM4Di–CNO groups (*n* = 7 animals/group). **(D)** Number of transitions between different pair of brain states during the light phase (ZT 0 –ZT 12) and dark phase (ZT 12 –ZT 24) of mice in CS+EGFP–CNO and CS+hM4Di–CNO groups (*n* = 7 animals/group). For all figures: one–way ANOVA with *Sidak’s* multiple comparisons test, *, *P* < 0.05; **, *P* < 0.001; ***, *P* < 0.0001; ns = no significant difference. Error bars indicate the SEM. Underlying data can be found in S1 Data.
(TIF)

S7 Fig The effects of activation of LHb–projecting vLGN/IGL neurons on NREM sleep. **(A)** Specific labeling of LHb–projecting vLGN/IGL neurons with hM3Dq–EGFP or EGFP. **(B)** LHb–projecting vLGN/IGL neurons expressing hM3Dq can be activated by bath application of CNO (10 μM, 100 s). **(C)** Schematic of the experimental design. **(D)** Representative EEG spectrograms, EEG and EMG traces (recorded from ZT 1 to ZT 2) of mice in different experimental groups. All animals received LHb injection of AAV2/2–Retro–Cre and i.p. injection of CNO (1 mg/kg). EGFP–CNO: mice that received vLGN/IGL injection of AAV2/9–DIO–EGFP; hM3Dq–CNO: mice that received vLGN/IGL injection of AAV2/9–DIO–hM3Dq–EGFP. **(E)** Left: time course changes of NREM sleep of mice in EGFP–CNO and hM3Dq–CNO groups (*n* = 6 animals/group). Middle: total NREM sleep amounts during the whole day (24 h) of mice in EGFP–CNO and hM3Dq–CNO groups. Right: NREM sleep amounts during light phase (ZT 0 –ZT 12) and dark phase (ZT 12 –ZT 24) of mice in EGFP–CNO and hM3Dq–CNO groups. **(F)** Number of transitions between different pair of brain states during the whole day (24 h) of mice in EGFP–CNO and hM3Dq–CNO groups (*n* = 6 animals/group). **(G)** Schematic of the experimental design. **(H)** Representative EEG spectrograms, EEG and EMG traces (recorded from ZT 1 to ZT 2) of mice in different experimental groups. All animals received LHb injection of AAV2/2–Retro–Cre, exposure to chronic stress and i.p. injection of CNO (1 mg/kg). CS+EGFP–CNO: mice that received vLGN/IGL injection of AAV2/9–DIO–EGFP; CS+hM3Dq–CNO: mice that received vLGN/IGL injection of AAV2/9–DIO–hM3Dq–EGFP. **(I)** Left: time course changes of NREM sleep of mice in CS+EGFP–CNO and CS+hM3Dq–CNO groups (*n* = 6 animals/group). Middle: total NREM sleep amounts during the whole day (24 h) of mice in CS+EGFP–CNO and CS+hM3Dq–CNO groups. Right: NREM sleep amounts during light phase (ZT 0 –ZT 12) and dark phase (ZT 12 –ZT 24) of mice in CS+EGFP–CNO and CS+hM3Dq–CNO groups. **(J)** Number of transitions between different pair of brain states during the whole day (24 h) of mice in CS+EGFP–CNO and CS+hM3Dq–CNO groups (*n* = 6 animals/group). For all figures: one–way ANOVA with *Sidak’s* multiple comparisons test, *, *P* < 0.05; **, *P* < 0.001; ns = no significant difference. Error bars indicate the SEM. Underlying data can be found in S1 Data.
(TIF)

S8 Fig Activation of the vLGN/IGL–LHb–RMTg pathway is required for the ability of bright light treatment to reduce the wakefulness–reducing effects of chronic stress. **(A)** Left: time course changes of REM sleep and wakefulness of mice in different experimental groups. All animals received exposure to CS, LT, LHb injection of rAAV2/2–Retro–Cre and i.p. injection of CNO (1 mg/kg). CS+LT+EGFP–CNO (*n* = 6 animals): mice that received vLGN/IGL injection of AAV2/9–DIO–EGFP; CS+LT+hM4Di–CNO (*n* = 7 animals): mice that received vLGN/IGL injection of AAV2/9–DIO–hM4Di–EGFP. Middle: total REM sleep and wakefulness amounts during the whole day (24 h) of mice in CS+LT+EGFP–CNO (*n* = 6 animals) and CS+LT+hM4Di–CNO (*n* = 7 animals) groups. Right: REM sleep and wakefulness amounts during the whole day (24 h), light phase (ZT 0 –ZT 12) and dark phase (ZT 12 –ZT 24) of mice in CS+LT+EGFP–CNO (*n* = 6 animals) and CS+LT+hM4Di–CNO (*n* = 7 animals) groups. **(B)** Number of transitions between different pair of brain states during the light phase (ZT 0 –ZT 12) and dark phase (ZT 12 –ZT 24) of animals in CS+LT+EGFP–CNO (*n* = 6 animals) and CS+LT+hM4Di–CNO (*n* = 7 animals) groups. W–N: Wake to NREM; N–W: NREM to Wake; N–R: NREM to REM. **(C)** Left: time course changes of REM sleep and wakefulness of mice in different experimental groups. All animals received exposure to chronic stress stimuli (CS), bright light treatment (LT), RMTg injection of rAAV2/2–Retro–Cre and i.p. injection of CNO (1 mg/kg) (*n* = 6 animals/group). CS+LT+EGFP–CNO: mice that received LHb injection of AAV2/9–DIO–EGFP. CS+LT+hM3Dq–CNO: mice that received LHb injection of AAV2/9–DIO–hM3Dq–EGFP. Middle: total REM sleep and wakefulness amounts during the whole day (24 h) of mice in CS+LT+EGFP–CNO and CS+LT+hM3Dq–CNO groups (*n* = 6 animals/group). Right: REM sleep and wakefulness amounts during the whole day (24 h), light phase (ZT 0 –ZT 12) and dark phase (ZT 12 –ZT 24) of mice in CS+LT+EGFP–CNO and CS+LT+hM3Dq–CNO groups (*n* = 6 animals/group). **(D)** Number of transitions between different pair of brain states during the light phase (ZT 0 –ZT 12) and dark phase (ZT 12 –ZT 24) of animals in CS+LT+EGFP–CNO and CS+LT+hM3Dq–CNO groups (*n* = 6 animals/group). **(E)** Left: time course changes of REM sleep and wakefulness of mice in different experimental groups. All animals received exposure to CS, LT, LHb injection of AAV2/1–Cre and i.p. injection of CNO (1 mg/kg). CS+LT+EGFP–CNO (*n* = 6 animals): mice that received RMTg injection of AAV2/9–DIO–EGFP. CS+LT+hM3Dq–CNO (*n* = 7 animals): mice that received RMTg injection of AAV2/9–DIO–hM3Dq–EGFP. Middle: total REM sleep and wakefulness amounts during the whole day (24 h) of mice in CS+LT+EGFP–CNO (*n* = 6 animals) and CS+LT+hM3Dq–CNO (*n* = 7 animals) groups. Right: REM sleep and wakefulness amounts during the whole day (24 h), light phase (ZT 0 –ZT 12) and dark phase (ZT 12 –ZT 24) of mice in CS+LT+EGFP–CNO (*n* = 6 animals) and CS+LT+hM3Dq–CNO (*n* = 7 animals) groups. **(F)** Number of transitions between different pair of brain states during the light phase (ZT 0 –ZT 12) and dark phase (ZT 12 –ZT 24) of animals in CS+LT+EGFP–CNO (*n* = 6 animals) and CS+LT+hM3Dq–CNO (*n* = 7 animals) groups. For all figures: one–way ANOVA with *Sidak’s* multiple comparisons test, *, *P* < 0.05; **, *P* < 0.001; ***, *P* < 0.0001; ns = no significant difference. Error bars indicate the SEM. Underlying data can be found in S1 Data.
(TIF)

S9 Fig The schematic of the visual circuit for the effects of bright light treatment on sleep alterations induced by chronic stress. (TIF)

S10 Fig Injection site verification. **(A)** Cocaine (10 mg/kg, i.p.) induced c–Fos expression in the VTA and RMTg. **(B)** A representative example showing RMTg postsynaptic neurons expressing hM3Dq–EGFP, with numbers indicating the distance from bregma. **(C)** A representative example of the location of the optical fiber employed to record the Ca^2+^ signals in RMTg neurons that received direct inputs from the LHb. **(D)** A representative example of the location of the injection site of rAAV2/2–Retro–Cre (visualized by CTB–647). Scale bars: 200 μm (A).
(TIF)

S1 Data Source data underlying the figures. (XLSX)

## Abbreviations

ACSF: artificial cerebrospinal fluid
DRN: dorsal raphe nucleus
EEG: electroencephalography
EMG: electromyography
EPSC: excitatory postsynaptic current
FST: forced swimming test
GPi: globus pallidus
IGL: intergeniculate leaflet
LH: lateral hypothalamus
LHb: lateral habenula
NA: numerical aperture
NREM: nonrapid eye movement
OFT: open field test
pHb: perihabenular nucleus
REM: rapid eye movement
RMTg: rostromedial tegmental nucleus
SPT: sucrose preference test
TST: tail suspension test
vLGN: ventral lateral geniculate nucleus
VP: ventral pallidum
VTA: ventral tegmental area
WRT: wheel-running test
ZT: Zeitgeber time
