# Supplementary figures and images for: Bright light treatment counteracts stress-induced sleep alterations in mice, via a visual circuit related to the rostromedial tegmental nucleus
Source: PLoS Biol. 2023 Sep 7;21(9):e3002282. doi: 10.1371/journal.pbio.3002282 (PMC10484455; doi:10.1371/journal.pbio.3002282)

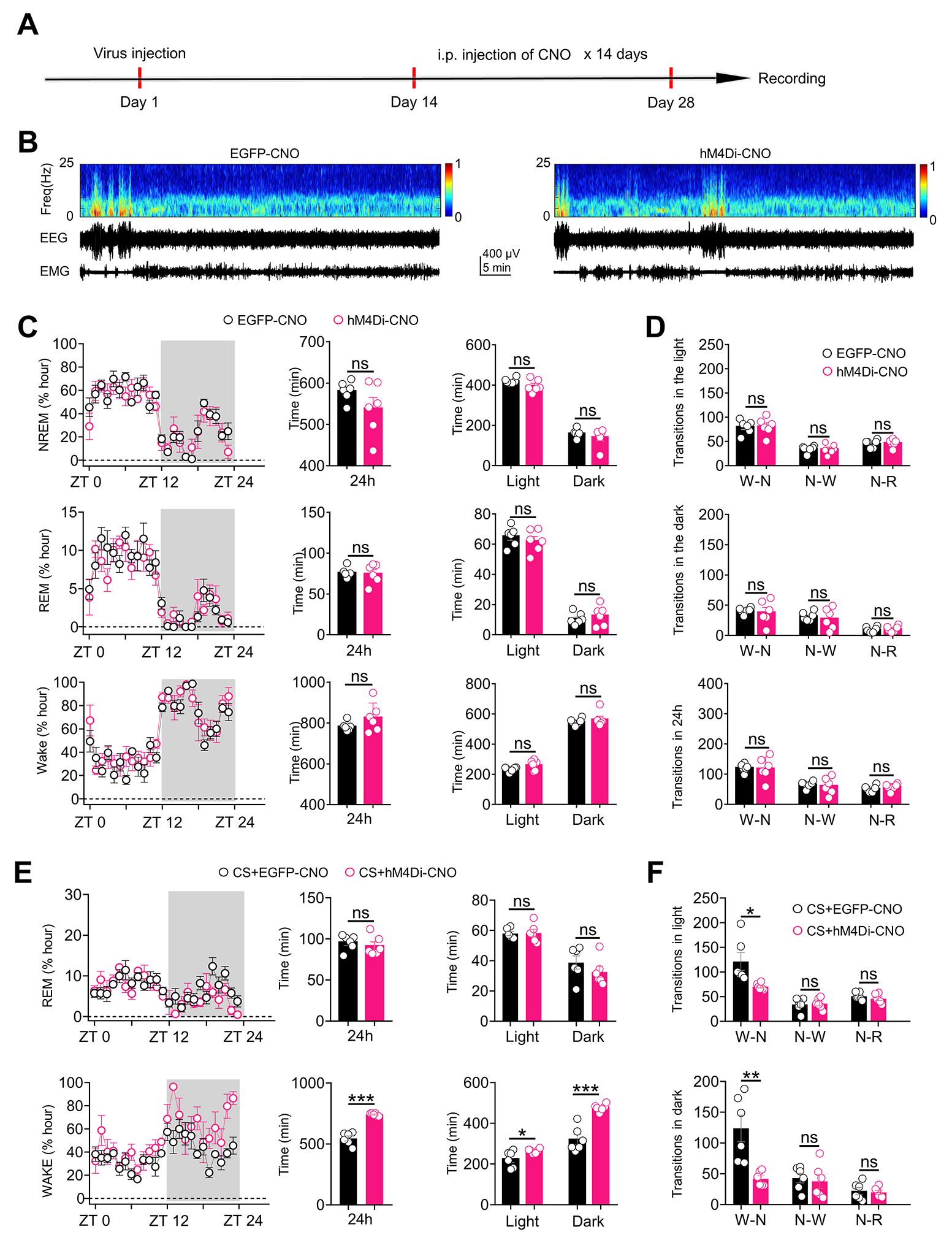

Supplement: S1 Fig — (A) Schematic of the experimental design. (B) Representative EEG spectrograms, EEG and EMG traces (recorded from ZT 1 to ZT 2) of mice in different experimental groups. All animals received i.p. injection of CNO (1 mg/kg). EGFP–CNO: mice that received LHb injection of AAV2/9–hSyn–EGFP; hM4Di–CNO: mice that received LHb injection of AAV2/9–hSyn–hM4Di–EGFP. (C) Left: time course changes of NREM and REM sleep and wakefulness of mice in EGFP–CNO and hM4Di–CNO groups (n = 6 animals/group). Middle: total sleep–wake amounts during the whole day (24 h) of mice in in EGFP–CNO and hM4Di–CNO groups. Right: total sleep–wake amounts during light phase (ZT 0 –ZT 12) and dark phase (ZT 12 –ZT 24) of mice in in EGFP–CNO and hM4Di–CNO groups. (D) Number of transitions between different pair of brain states during the light phase (ZT 0 –ZT 12), dark phase (ZT 12 –ZT 24), and the whole day (24 h) of mice in EGFP–CNO and hM4Di–CNO groups (n = 6 animals/group). W–N: Wake to NREM; N–W: NREM to Wake; N–R: NREM to REM. (E) Left: time course changes of REM sleep and wakefulness of mice in different experimental groups (n = 6 animals/group). All animals received exposure to chronic stress stimuli (CS) and i.p. injection of CNO (1 mg/kg). CS+EGFP–CNO: mice that received LHb injection of AAV2/9–hSyn–EGFP. CS+hM4Di–CNO: mice that received LHb injection of AAV2/9–hSyn–hM4Di–EGFP. Middle: total REM sleep and wakefulness amounts during the whole day (24 h) of mice in CS+EGFP–CNO and CS+hM4Di–CNO groups (n = 6 animals/group). Right: REM sleep and wakefulness amounts during light phase (ZT 0 –ZT 12) and dark phase (ZT 12 –ZT 24) of mice in CS+EGFP–CNO and CS+hM4Di–CNO groups (n = 6 animals/group). (F) Number of transitions between different pair of brain states during the light phase (ZT 0 –ZT 12) and dark phase (ZT 12 –ZT 24) of animals in CS+EGFP–CNO and CS+hM4Di–CNO groups (n = 6 animals/group). W–N: Wake to NREM; N–W: NREM to Wake; N–R: NREM to REM. For all figures: one–way ANOVA with Sidak’s mu [file pbio.3002282.s001.tif]

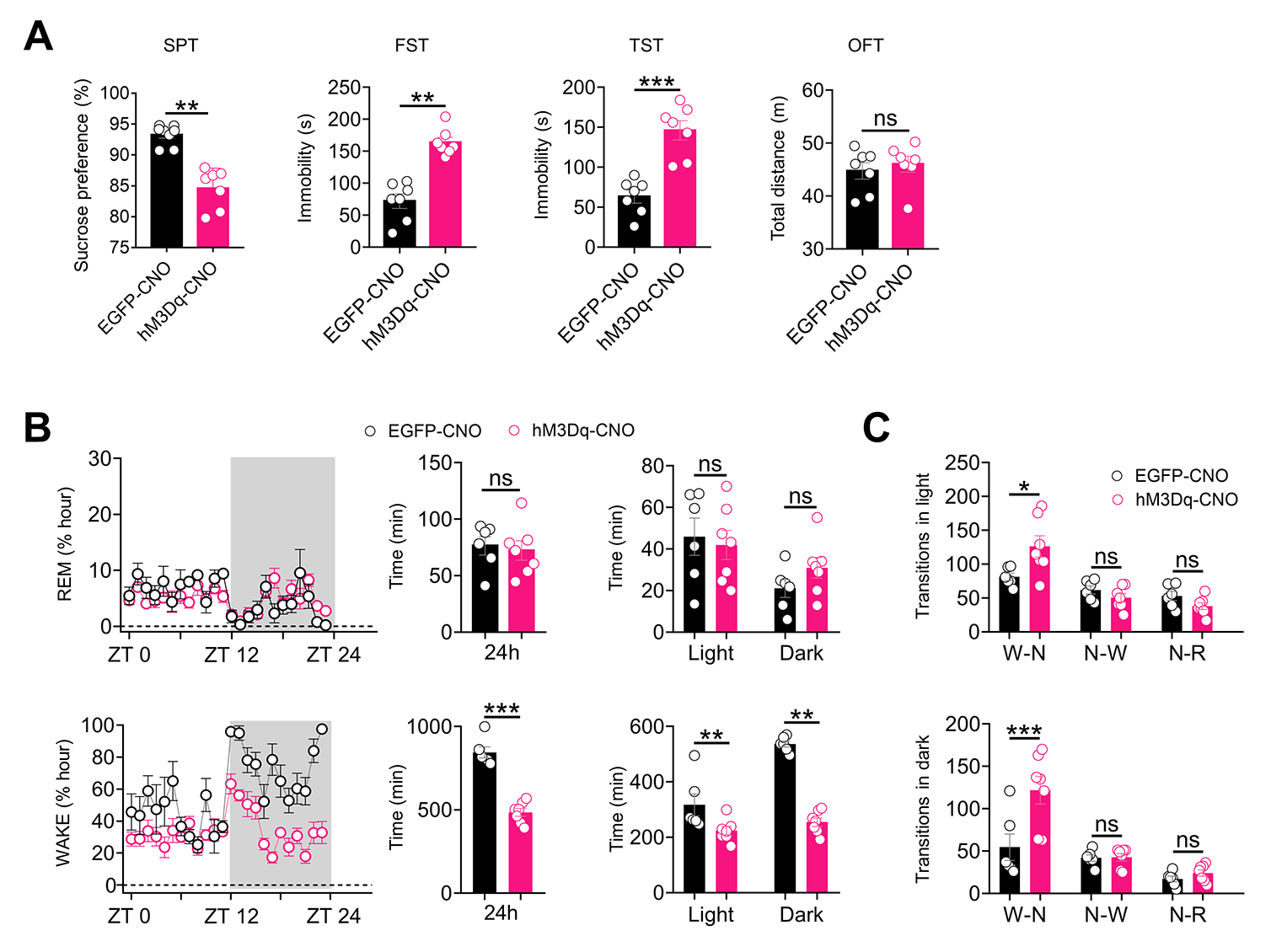

Supplement: S2 Fig — (A) Depressive–like behaviors in different experimental groups (n = 7 animals/group). All animals received i.p. injection of CNO (1 mg/kg). EGFP–CNO: mice that received LHb injection of AAV2/9–hSyn–EGFP; hM3Dq–CNO: mice that received LHb injection of AAV2/9–hSyn–hM3Dq–EGFP. (B) Left: time course changes of REM sleep and wakefulness of mice in in different experimental groups. All animals received i.p. injection of CNO (1 mg/kg). EGFP–CNO (n = 6 animals): mice that received LHb injection of AAV2/9–hSyn–EGFP. hM3Dq–CNO (n = 7 animals): mice that received LHb injection of AAV2/9–hSyn–hM3Dq–EGFP; Middle: total REM sleep and wakefulness amounts during the whole day (24 h) of mice in EGFP–CNO (n = 6 animals) and hM3Dq–CNO (n = 7 animals) groups. Right: REM sleep and wakefulness amounts during light phase (ZT 0 –ZT 12) and dark phase (ZT 12 –ZT 24) of mice in EGFP–CNO (n = 6 animals) and hM3Dq–CNO (n = 7 animals) groups. (C) Number of transitions between different pair of brain states during the light phase (ZT 0 –ZT 12) and dark phase (ZT 12 –ZT 24) of mice in EGFP–CNO (n = 6 animals) and hM3Dq–CNO (n = 7 animals) groups. For all figures: one–way ANOVA with Sidak’s multiple comparisons test, *, P < 0.05; **, P < 0.001; ***, P < 0.0001; ns = no significant difference. Error bars indicate the SEM. Underlying data can be found in S1 Data. (TIF) [file pbio.3002282.s002.tif]

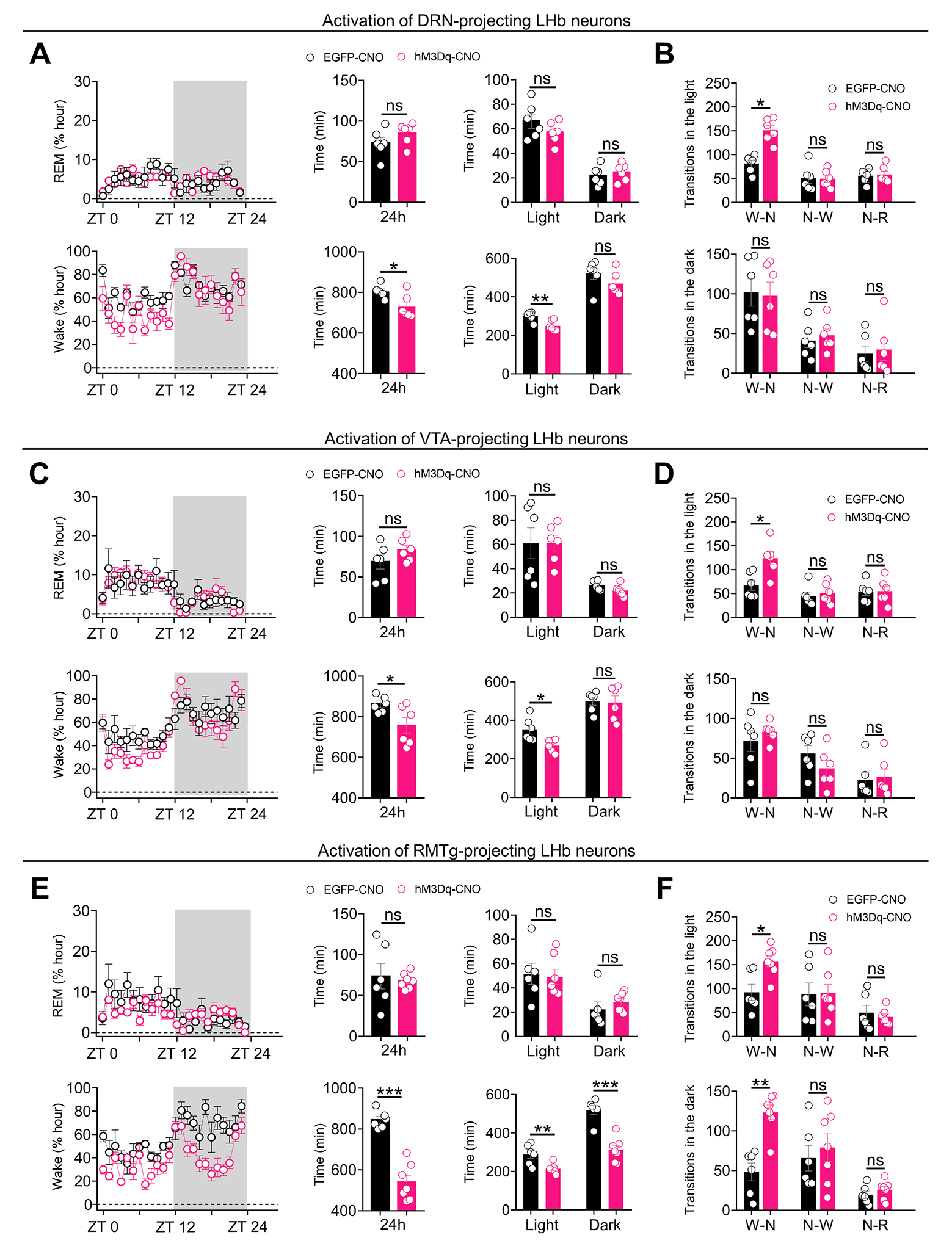

Supplement: S3 Fig — (A, C, E) Left: time course changes of REM sleep and wakefulness of mice in different experimental groups. All mice received i.p. injection of CNO (1 mg/kg). EGFP–CNO (n = 6 animals/group): mice that received DRN (A) or VTA (C) or RMTg (E) injection of rAAV2/2–Retro–Cre and LHb injection of AAV2/9–DIO–EGFP; hM3Dq–CNO: mice that received DRN (A, n = 6 animals) or VTA (C, n = 6 animals) or RMTg (E, n = 7 animals) injection of rAAV2/2–Retro–Cre and LHb injection of AAV2/9–DIO–hM3Dq–EGFP. Right: REM sleep and wakefulness amounts during the whole day (24 h), light phase (ZT 0 –ZT 12) and dark phase (ZT 12 –ZT 24) of mice EGFP–CNO and hM3Dq–CNO groups. (B, D, F) Number of transitions between different pair of brain states during the light phase (ZT 0 –ZT 12) and dark phase (ZT 12 –ZT 24) of mice in EGFP–CNO and hM3Dq–CNO groups. W–N: Wake to NREM; N–W: NREM to Wake; N–R: NREM to REM. For all figures: one–way ANOVA test, *, P < 0.05; **, P < 0.01; ***, P < 0.0001; ns = no significant difference. Error bars indicate the SEM. Underlying data can be found in S1 Data. (TIF) [file pbio.3002282.s003.tif]

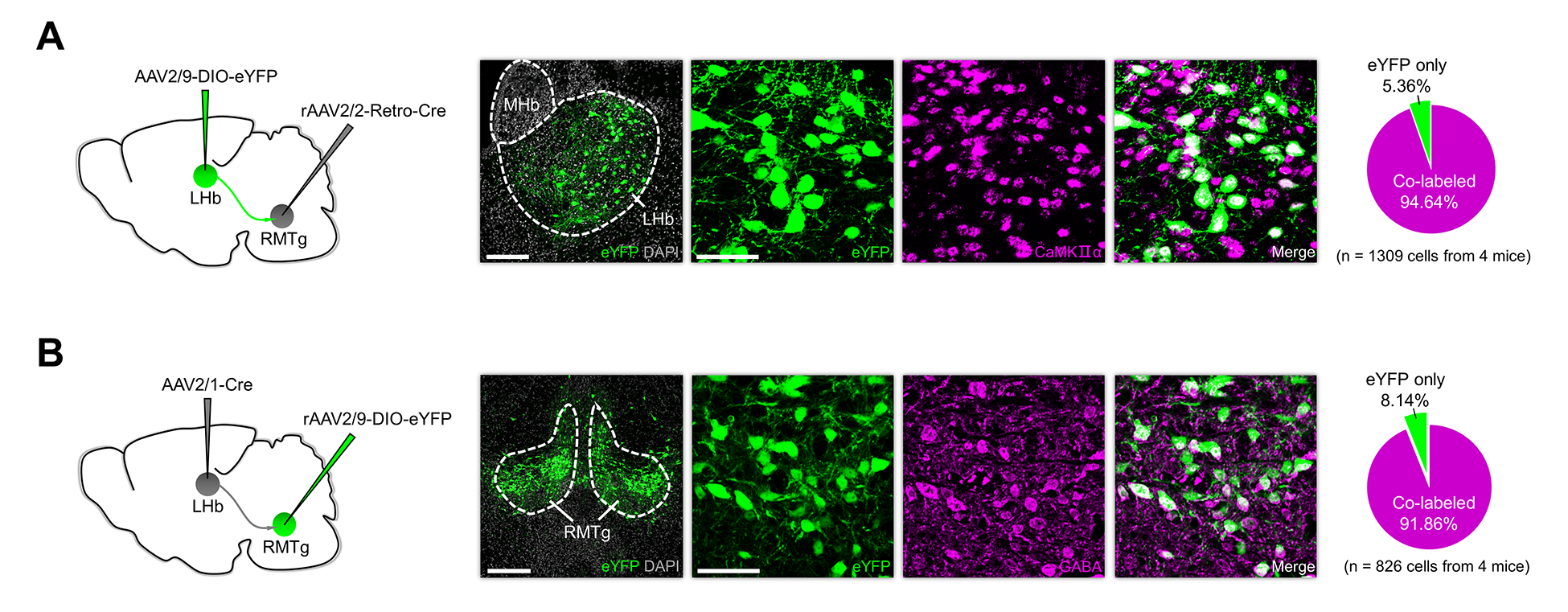

Supplement: S4 Fig — (A) Left: scheme for specific labeling of RMTg–projecting LHb neurons with eYFP. Middle: representative images of the LHb illustrating eYFP–expressing LHb neurons co–labeled with CaMKIIα. Right: pie chart indicates percentage of eYFP–expressing LHb neurons co–labeled with CaMKIIα. (B) Left: scheme for specific labeling of RMTg neurons receiving direct LHb inputs with eYFP. Middle: representative images of the RMTg illustrating eYFP–expressing RMTg neurons co–labeled with GABA. Right: pie chart indicates percentage of eYFP–expressing RMTg neurons co–labeled with GABA. Scale bars: 200 μm (A–left, B–left); 50 μm (A–right, B–right). Underlying data can be found in S1 Data. (TIF) [file pbio.3002282.s004.tif]

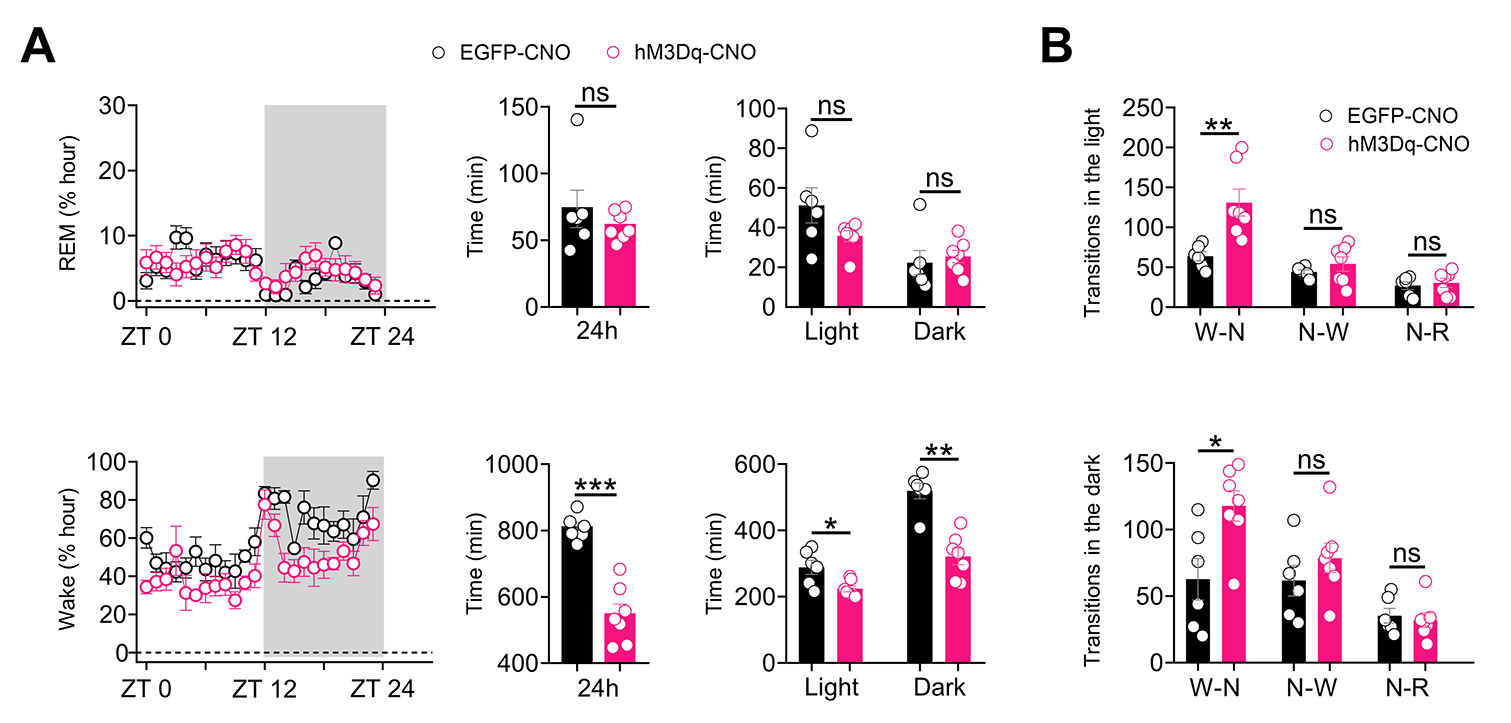

Supplement: S5 Fig — (A) Left: time course changes of REM sleep and wakefulness of mice in different experimental groups. All mice received LHb injection of AAV2/1–Cre and i.p. injection of CNO (1 mg/kg). EGFP–CNO (n = 6 animals): mice that received RMTg injection of AAV2/9–DIO–EGFP. hM3Dq–CNO (n = 7 animals): mice that received RMTg injection of AAV2/9–DIO–hM3Dq–EGFP. Middle: total REM sleep and wakefulness amounts during the whole day (24 h) of mice in n EGFP–CNO (n = 6 animals) and hM3Dq–CNO groups (n = 7 animals). Right: REM sleep and wakefulness amounts during light phase (ZT 0 –ZT 12) and dark phase (ZT 12 –ZT 24) of mice in n EGFP–CNO (n = 6 animals) and hM3Dq–CNO (n = 7 animals) groups. (B) Number of transitions between different pair of brain states during the light phase (ZT 0 –ZT 12) and dark phase (ZT 12 –ZT 24) of mice in EGFP–CNO (n = 6 animals) and hM3Dq–CNO (n = 7 animals) groups. W–N: Wake to NREM; N–W: NREM to Wake; N–R: NREM to REM. For all figures: one–way ANOVA test, *, P < 0.05; **, P < 0.01; ***, P < 0.0001; ns = no significant difference. Error bars indicate the SEM. Underlying data can be found in S1 Data. (TIF) [file pbio.3002282.s005.tif]

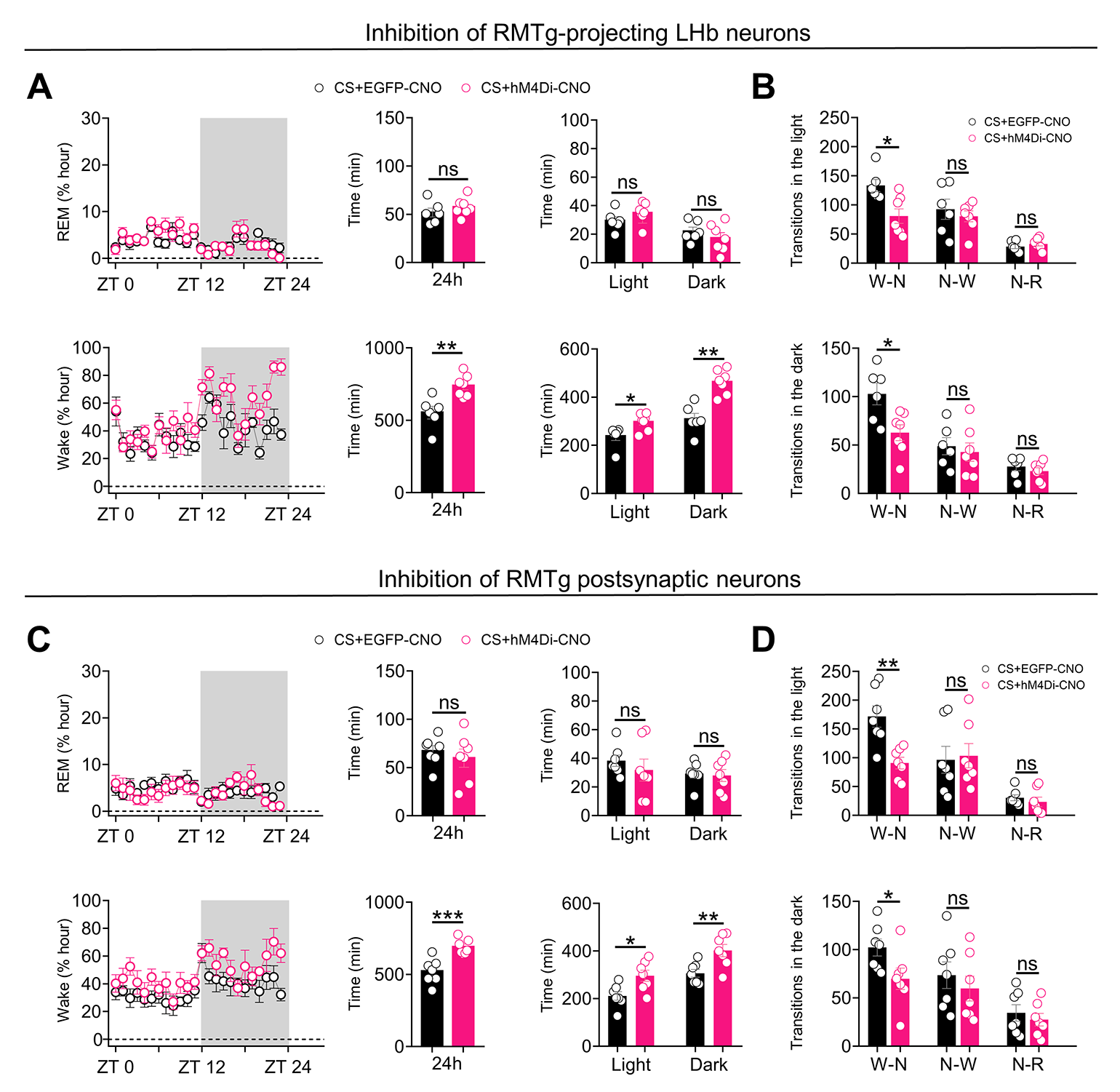

Supplement: S6 Fig — (A) Left: time course changes of REM sleep and wakefulness of mice in different experimental groups. All animals received exposure to chronic stress stimuli (CS), RMTg injection of rAAV2/2–Retro–Cre and i.p injection of CNO (1 mg/kg). CS+EGFP–CNO (n = 6 animals): mice that received LHb injection of AAV2/9–DIO–EGFP. CS+hM4Di–CNO (n = 7 animals): mice that received LHb injection of AAV2/9–DIO–hM4Di–EGFP. Middle: total REM sleep and wakefulness amounts during the whole day (24 h) of mice in CS+EGFP–CNO (n = 6 animals) and CS+hM4Di–CNO (n = 7 animals) groups. Right: REM sleep and wakefulness amounts during the whole day (24 h), light phase (ZT 0 –ZT 12) and dark phase (ZT 12 –ZT 24) of mice in CS+EGFP–CNO (n = 6 animals) and CS+hM4Di–CNO (n = 7 animals) groups. (B) Number of transitions between different pair of brain states during the light phase (ZT 0 –ZT 12) and dark phase (ZT 12 –ZT 24) of mice in CS+EGFP–CNO (n = 6 animals) and CS+hM4Di–CNO (n = 7 animals) groups. W–N: Wake to NREM; N–W: NREM to Wake; N–R: NREM to REM. (C) Left: time course changes of REM sleep and wakefulness of mice in different experimental groups (n = 7 animals/group). All animals received exposure to CS, LHb injection of AAV2/1–Cre and i.p. injection of CNO (1 mg/kg). CS+EGFP–CNO: mice that received RMTg injection of AAV2/9–DIO–EGFP. CS+hM4Di–CNO: mice that received RMTg injection of AAV2/9–DIO–hM4Di–EGFP. Middle: total REM sleep and wakefulness amounts during the whole day (24 h) of mice in CS+EGFP–CNO and CS+hM4Di–CNO groups (n = 7 animals/group). Right: REM sleep and wakefulness amounts during the whole day (24 h), light phase (ZT 0 –ZT 12) and dark phase (ZT 12 –ZT 24) of mice in CS+EGFP–CNO and CS+hM4Di–CNO groups (n = 7 animals/group). (D) Number of transitions between different pair of brain states during the light phase (ZT 0 –ZT 12) and dark phase (ZT 12 –ZT 24) of mice in CS+EGFP–CNO and CS+hM4Di–CNO groups (n = 7 animals/group). For all figures: one–way ANOVA with Sidak’s multiple c [file pbio.3002282.s006.tif]

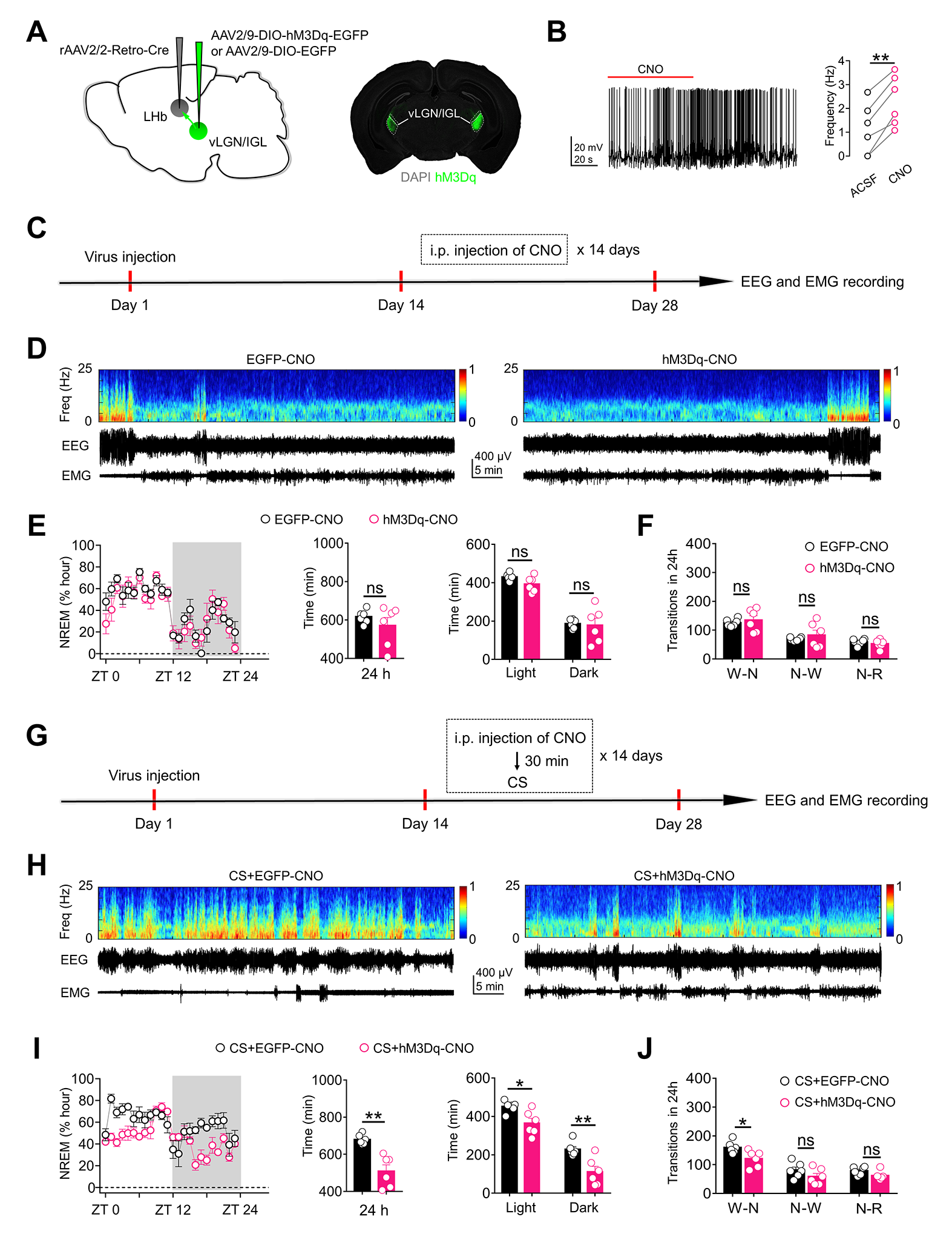

Supplement: S7 Fig — (A) Specific labeling of LHb–projecting vLGN/IGL neurons with hM3Dq–EGFP or EGFP. (B) LHb–projecting vLGN/IGL neurons expressing hM3Dq can be activated by bath application of CNO (10 μM, 100 s). (C) Schematic of the experimental design. (D) Representative EEG spectrograms, EEG and EMG traces (recorded from ZT 1 to ZT 2) of mice in different experimental groups. All animals received LHb injection of AAV2/2–Retro–Cre and i.p. injection of CNO (1 mg/kg). EGFP–CNO: mice that received vLGN/IGL injection of AAV2/9–DIO–EGFP; hM3Dq–CNO: mice that received vLGN/IGL injection of AAV2/9–DIO–hM3Dq–EGFP. (E) Left: time course changes of NREM sleep of mice in EGFP–CNO and hM3Dq–CNO groups (n = 6 animals/group). Middle: total NREM sleep amounts during the whole day (24 h) of mice in EGFP–CNO and hM3Dq–CNO groups. Right: NREM sleep amounts during light phase (ZT 0 –ZT 12) and dark phase (ZT 12 –ZT 24) of mice in EGFP–CNO and hM3Dq–CNO groups. (F) Number of transitions between different pair of brain states during the whole day (24 h) of mice in EGFP–CNO and hM3Dq–CNO groups (n = 6 animals/group). (G) Schematic of the experimental design. (H) Representative EEG spectrograms, EEG and EMG traces (recorded from ZT 1 to ZT 2) of mice in different experimental groups. All animals received LHb injection of AAV2/2–Retro–Cre, exposure to chronic stress and i.p. injection of CNO (1 mg/kg). CS+EGFP–CNO: mice that received vLGN/IGL injection of AAV2/9–DIO–EGFP; CS+hM3Dq–CNO: mice that received vLGN/IGL injection of AAV2/9–DIO–hM3Dq–EGFP. (I) Left: time course changes of NREM sleep of mice in CS+EGFP–CNO and CS+hM3Dq–CNO groups (n = 6 animals/group). Middle: total NREM sleep amounts during the whole day (24 h) of mice in CS+EGFP–CNO and CS+hM3Dq–CNO groups. Right: NREM sleep amounts during light phase (ZT 0 –ZT 12) and dark phase (ZT 12 –ZT 24) of mice in CS+EGFP–CNO and CS+hM3Dq–CNO groups. (J) Number of transitions between different pair of brain states during the whole day (24 h) of mice in [file pbio.3002282.s007.tif]

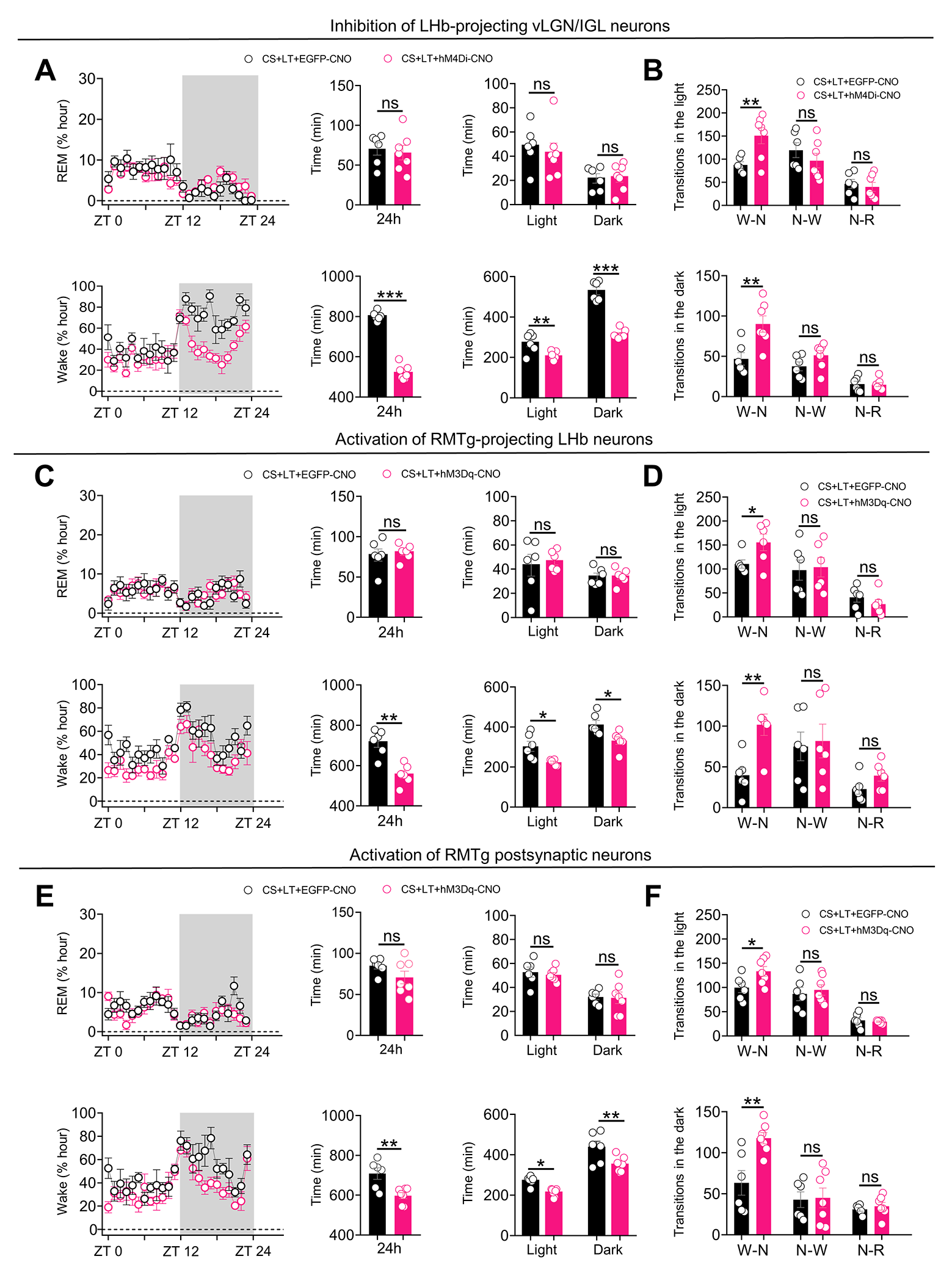

Supplement: S8 Fig — (A) Left: time course changes of REM sleep and wakefulness of mice in different experimental groups. All animals received exposure to CS, LT, LHb injection of rAAV2/2–Retro–Cre and i.p. injection of CNO (1 mg/kg). CS+LT+EGFP–CNO (n = 6 animals): mice that received vLGN/IGL injection of AAV2/9–DIO–EGFP; CS+LT+hM4Di–CNO (n = 7 animals): mice that received vLGN/IGL injection of AAV2/9–DIO–hM4Di–EGFP. Middle: total REM sleep and wakefulness amounts during the whole day (24 h) of mice in CS+LT+EGFP–CNO (n = 6 animals) and CS+LT+hM4Di–CNO (n = 7 animals) groups. Right: REM sleep and wakefulness amounts during the whole day (24 h), light phase (ZT 0 –ZT 12) and dark phase (ZT 12 –ZT 24) of mice in CS+LT+EGFP–CNO (n = 6 animals) and CS+LT+hM4Di–CNO (n = 7 animals) groups. (B) Number of transitions between different pair of brain states during the light phase (ZT 0 –ZT 12) and dark phase (ZT 12 –ZT 24) of animals in CS+LT+EGFP–CNO (n = 6 animals) and CS+LT+hM4Di–CNO (n = 7 animals) groups. W–N: Wake to NREM; N–W: NREM to Wake; N–R: NREM to REM. (C) Left: time course changes of REM sleep and wakefulness of mice in different experimental groups. All animals received exposure to chronic stress stimuli (CS), bright light treatment (LT), RMTg injection of rAAV2/2–Retro–Cre and i.p. injection of CNO (1 mg/kg) (n = 6 animals/group). CS+LT+EGFP–CNO: mice that received LHb injection of AAV2/9–DIO–EGFP. CS+LT+hM3Dq–CNO: mice that received LHb injection of AAV2/9–DIO–hM3Dq–EGFP. Middle: total REM sleep and wakefulness amounts during the whole day (24 h) of mice in CS+LT+EGFP–CNO and CS+LT+hM3Dq–CNO groups (n = 6 animals/group). Right: REM sleep and wakefulness amounts during the whole day (24 h), light phase (ZT 0 –ZT 12) and dark phase (ZT 12 –ZT 24) of mice in CS+LT+EGFP–CNO and CS+LT+hM3Dq–CNO groups (n = 6 animals/group). (D) Number of transitions between different pair of brain states during the light phase (ZT 0 –ZT 12) and dark phase (ZT 12 –ZT 24) of animals in CS+LT+EGFP–CNO a [file pbio.3002282.s008.tif]

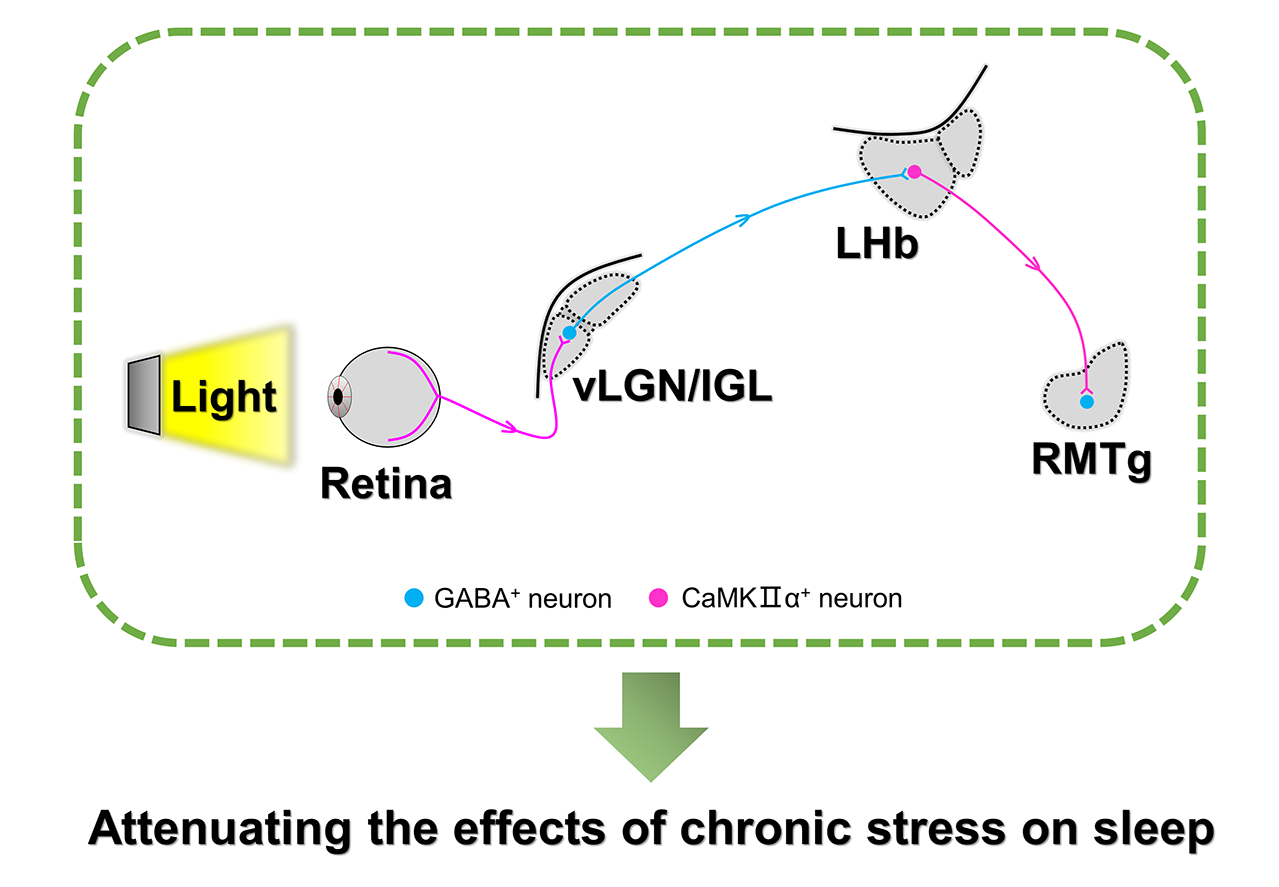

Supplement: S9 Fig — (TIF) [file pbio.3002282.s009.tif]

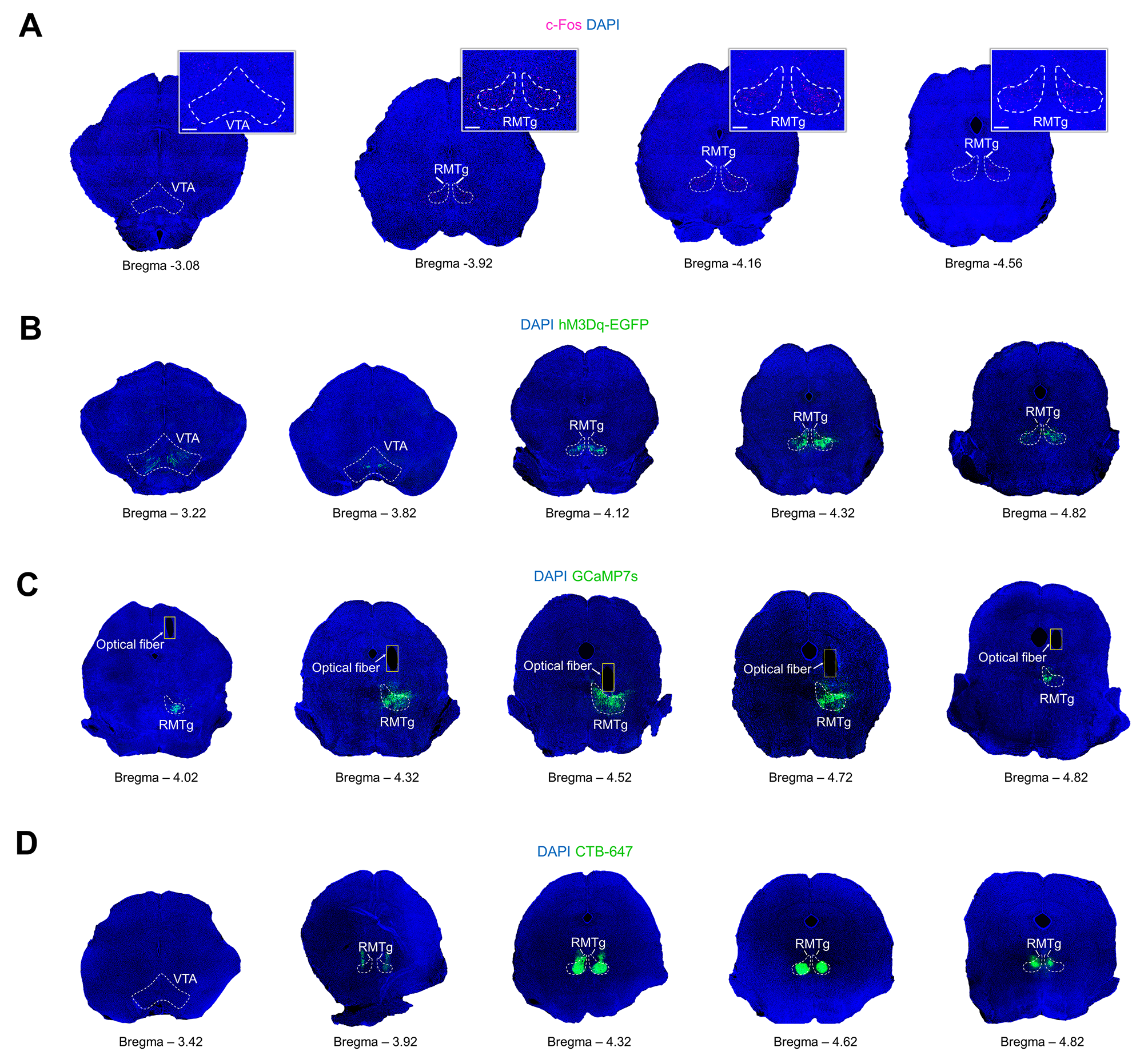

Supplement: S10 Fig — (A) Cocaine (10 mg/kg, i.p.) induced c–Fos expression in the VTA and RMTg. (B) A representative example showing RMTg postsynaptic neurons expressing hM3Dq–EGFP, with numbers indicating the distance from bregma. (C) A representative example of the location of the optical fiber employed to record the Ca2+ signals in RMTg neurons that received direct inputs from the LHb. (D) A representative example of the location of the injection site of rAAV2/2–Retro–Cre (visualized by CTB–647). Scale bars: 200 μm (A). (TIF) [file pbio.3002282.s010.tif]
